# Epigenetics and stroke: role of DNA methylation and effect of aging on blood–brain barrier recovery

**DOI:** 10.1186/s12987-023-00414-7

**Published:** 2023-02-28

**Authors:** Chelsea M. Phillips, Svetlana M. Stamatovic, Richard F. Keep, Anuska V. Andjelkovic

**Affiliations:** 1 Department of Pathology, Medical School, University of Michigan, 7520A MSRB I, 1150 W Medical Center Dr, Ann Arbor, MI 48109-5602 USA; 2 Department of Neurosurgery, Medical School, University of Michigan, 7520A MSRB I, 1150 W Medical Center Dr, Ann Arbor, MI 48109-5602 USA; 3 Department of Molecular and Integrative Physiology, University of Michigan, Ann Arbor, MI USA; 4 Neuroscience Graduate Program, University of Michigan, Ann Arbor, MI USA

**Keywords:** Blood–brain barrier, Brain repair, DNA methylation, Epigenetics, Stroke, Transcriptomics, Aging

## Abstract

Incomplete recovery of blood–brain barrier (BBB) function contributes to stroke outcomes. How the BBB recovers after stroke remains largely unknown. Emerging evidence suggests that epigenetic factors play a significant role in regulating post-stroke BBB recovery. This study aimed to evaluate the epigenetic and transcriptional profile of cerebral microvessels after thromboembolic (TE) stroke to define potential causes of limited BBB recovery. RNA-sequencing and reduced representation bisulfite sequencing (RRBS) analyses were performed using microvessels isolated from young (6 months) and old (18 months) mice seven days poststroke compared to age-matched sham controls. DNA methylation profiling of poststroke brain microvessels revealed 11,287 differentially methylated regions (DMR) in old and 9818 DMR in young mice, corresponding to annotated genes. These DMR were enriched in genes encoding cell structural proteins (e.g., cell junction, and cell polarity, actin cytoskeleton, extracellular matrix), transporters and channels (e.g., potassium transmembrane transporter, organic anion and inorganic cation transporters, calcium ion transport), and proteins involved in endothelial cell processes (e.g., angiogenesis/vasculogenesis, cell signaling and transcription regulation). Integrated analysis of methylation and RNA sequencing identified changes in cell junctions (occludin), actin remodeling (ezrin) as well as signaling pathways like Rho GTPase (RhoA and Cdc42ep4). Aging as a hub of aberrant methylation affected BBB recovery processes by profound alterations (hypermethylation and repression) in structural protein expression (e.g., claudin-5) as well as activation of a set of genes involved in endothelial to mesenchymal transformation (e.g., *Sox9*, *Snai1*), repression of angiogenesis and epigenetic regulation. These findings revealed that DNA methylation plays an important role in regulating BBB repair after stroke, through regulating processes associated with BBB restoration and prevalently with processes enhancing BBB injury.

**Supplementary Information:**

The online version contains supplementary material available at 10.1186/s12987-023-00414-7.

## Introduction

Stroke is a major cause of death and long-term disability, particularly in the elderly [1, 2]. The average stroke survival time is 6 to 7 years with many patients enduring physical disability and late cognitive impairment [3–5]. Neurological outcomes after stroke depend on numerous factors including age, infarct size and location, genetic factors, as well as the degree of brain repair [6–9]. Poststroke brain repair involves many events including reestablishing blood–brain barrier (BBB) structure and function [9–12].

BBB restoration after stroke is a tightly regulated process involving de novo synthesis of junctional proteins for regenerating barrier integrity, as well as rebuilding other BBB systems (e.g., transport) to institute BBB functionality [11, 13–16]. However, a growing body of clinical and experimental evidence indicates the BBB never fully recovers after stroke and that persistent BBB leakiness exists for days and months [14, 17–20]. Although small barrier leakage may be beneficial short-term through increases in waste product clearance and tissue nutrient supply, it is harmful in in the long-term. Often described as poststroke BBB dysfunction, this condition is characterized by junctional complex alterations, particularly tight junction (TJ) complex instability, induction of fluid-phase or nonspecific pinocytosis and transcytosis, formation of transendothelial channels, endothelial cell membrane disruption and intense neurovascular unit remodeling (activation of pericytes and astrocytes and vascular innervation) [12, 17, 21–24]. The consequences are chronic inflammation, secondary neuronal injury, and recurrent stroke [11, 25–27]. Although the potential causes of poststroke BBB leakiness/dysfunction are still largely unknown, incomplete angiogenesis, uncontrolled inflammation, and defects in structural repair are implied. Thus, identifying and characterizing the factors and processes that contribute to incomplete BBB recovery hold a key for successful BBB restoration and improving poststroke recovery.

Recently, epigenetics has emerged as an important contributor to stroke pathogenesis and post-stroke recovery, acting as a higher order regulatory mechanism of tissue repair. Defined as altered gene expression independent of primary changes to the DNA sequence, epigenetics depends upon interactions between environmental factors and the genome [28, 29]. Three epigenetic factors are essential for organism function: DNA methylation, post-translational histone modifications (e.g., acetylation, methylation, phosphorylation, and ubiquitination), and non-coding RNAs.

One of the best understood epigenetic processes is DNA methylation, which involves addition of methyl groups to cytosine residues, specifically those preceding guanine residues in CpG islands or CpG sites (defined as 500 bp in size, with a GC content > 55%) [30, 31]. DNA methyltransferases (DNMTs; including DNMT-1, -2, -3A, -3B, -3L) catalyze the covalent transfer of a methyl group from an S-adenosyl methionine to the cytosine residue [32, 33]. The DNA methylome is not only maintained by addition of methyl groups, but also through demethylation processes. DNA demethylation can be passive, via dysregulation of DNMT1 and passive incorporation of unmethylated cytosines into the genome, or active, through oxidation or deamination catalyzed by ten-eleven translocations (TETs) and activation-induced deaminase (AID) [27, 34, 35]. The relationship between gene expression and DNA methylation is best understood within the context of gene promoter methylation. Addition of methyl groups (hypermethylation) to CpG islands within gene promoters is associated with transcriptional repression, as it prevents transcription factors and RNA polymerase II from binding to DNA [29, 30, 36]. In contrast, promoter region hypomethylation is associated with transcriptional activation [27, 36]. Nevertheless, gene body methylation is less understood, and may result in transcriptional activation or repression, dependent on context.

Altered DNA methylation patterns are hallmarks of multitude diseases including cerebrovascular disease [37, 38]. For example, patients with a high risk for stroke occurrence have hypomethylated Long Interspersed Nucleotide Element-1 (LINE-1) repeats, associated with increased circulating vascular cell adhesion molecule-1 (VCAM-1) levels [39, 40]. Similarly, hypomethylation of TNF receptor-associated factor 3 (TRAF3), hypermethylation of thrombospondin-1 (THBS1), and increased DNA methyltransferase 3A (DNMT3A) activity are indicated as predictors of stroke outcome [41–43]. Regarding BBB and stroke, DNA methylation is implicated in BBB permeability regulation. Hypermethylation of the tissue inhibitor of metalloproteinase 2 (TIMP2) promoter decreases TIMP2 expression/activity, diminishing the balance with metalloproteinase 2 and 9 and causing TJ protein and extracellular matrix degradation [44]. Such limited findings suggest a role of DNA methylation in stroke and regulating poststroke BBB recovery. However, the link between DNA methylation and post-stroke BBB functional and structural integrity is still largely unknown.

The present study addresses the transcriptional and methylome landscapes of poststroke BBB recovery that could define the plasticity and capacity for BBB restoration after stroke. As aging has critical roles in stroke occurrence, outcomes and recovery, this study examines the methylome and transcriptome signatures of poststroke BBB recovery in young and old mice, highlighting age-related processes and factors that could affect capacity for BBB restoration.

## Methods

### Animals

All experimental procedures were approved by the Institutional Animal Care and Use Committee of the University of Michigan. Experiments used male C57BL/6 mice from the Jackson Laboratory (10–12 weeks of age) and aging C57BL/6 mice (18–20 months) from the National Institute of Aging colony.

### Thromboembolic stroke

Thromboembolic (TE) stroke was induced by injecting a platelet-rich microemboli suspension (particle size ~ 4 µm) as described previously [45, 46]. Briefly, a thromboemboli suspension was prepared by mixing arterial blood with thrombin solution [60 U thrombin (Sigma Aldrich, St Louis, MO USA) in 0.9% NaCl) at a ratio of 4:1. The thromboemboli suspension (8 mg/100 µl) was injected into the internal carotid artery. For the injection, mice were anesthetized by intraperitoneal injection of ketamine (100 mg/kg) and xylazine (10 mg/kg). During the surgery, core body temperature was maintained at 37 ± 0.5 °C. Sham-operated mice underwent all procedures except embolization.

### Neurological score

Stroke-related neurological deficits were scored as described by Yamamoto et al. [47] on days one and five post-surgery. Briefly, mice were scored on two parameters: body symmetry and forelimb symmetry. The grading was as follows: 0 = no deficit (no body twisting), 1 = mild deficit (asymmetric twisting tendency of the body), and 2 = severe deficit (consistently twisting). Limb motor function was graded: 0 = no deficit (no flexion of forelimbs), 1 = mild deficit (intermittent asymmetrical flexion of forelimbs), and 2 = severe deficit (forelimb flexion was consistent).

### Magnetic resonance imaging (MRI) analysis

MRI was performed on a 7.0T Agilent MR scanner (horizontal bore, Agilent, Palo Alto, CA, USA). Axial T2-weighted images were acquired using a fast spin-echo sequence with the following parameters: repetition time/effective echo time, 4000/60 ms; echo spacing, 15 ms; number of echoes, 8; field of view, 20 × 20 mm; matrix, 256 × 128; slice thickness, 0.5 mm; number of slices 25. For MRI analysis, all images were first evaluated for adequate signal-to-noise ratio, presence of significant motion or other artifacts, and consistency of the sequence parameters. Infarct size was analyzed using the image analysis software Image J (National Institute of Health, USA) [48, 49]. Briefly, hyperintense areas on each slice under constant contrast value were determined by computer-aided manual tracing and calculated by summing the volumes from each slide. For BBB permeability, an influx rate constant (*K*_*i*_; min^−1^) for Gd-DTPA (*ip* bolus injection 100 ml/ 0.5 mM, BioPAL, Worchester, MA) was calculated using the Patlak model and established protocols [50, 51].

### Microvessel isolation

Brain microvessels were isolated from the ischemic hemispheres of young and old C57BL/6 mice at 7 days post-TE stroke, as well as corresponding hemispheres in sham operated mice using an established protocol [17, 52]. Briefly, brain tissue was minced in Dulbecco’s Phosphate Buffered Saline (DPBS, Life Technology Corporation, Grand Island, NY USA) and homogenized gently in a Dounce glass homogenizer. Myelin was removed by centrifugation in a 15% Dextran solution (Dextran MW ∼70,000, Sigma Aldrich, St Louis, MO USA). The obtained pellet was transferred to 40 mm cell strainer and washed by DPBS supplemented by 0.5% endotoxin-, fatty acid- and protease free bovine serum albumin (BSA, Sigma-Aldrich, St Louis MO USA) to retrieve microvessels. The isolated blood vessels were then digested for 5 min with 0.25% trypsin (Life Technology Corporation, Grand Island, NY USA) at 37 °C to remove perivascular cells. Microvessels’ purity was evaluated by immunocytochemistry using anti-CD31 (brain endothelial cells; BD Bioscience), GFAP (astrocytes; Sigma Aldrich, St Louis, MO USA), PDGFRβ (pericytes; Abcam, Waltham, MA, USA), and Iba1 (microglia; Abcam, Waltham, MA, USA) antibodies. The protocol produced 99.99% “clean” (without perivascular cells) blood vessels. Isolated brain microvessels were further processed for paired-end mRNA-sequencing and reduced representation bisulfite sequencing for transcriptome and DNA methylome analyses, respectively.

### Global methylation assay

Global DNA methylation of genomic DNA from brain microvessels was measured using the Global DNA Methylation LINE-1 Kit (Diagenode Inc. Denville, NJ). Assay and data analysis were conducted according to the manufacturer’s protocol, with analysis measuring the percent of 5-methylcytosines (% 5-mC) based on CpG residues.

### Reduced representation bisulfite sequencing (RRBS)

Genomic DNA was isolated from brain microvessels using the DNeasy Blood & Tissue Kit (Qiagen). DNA concentration was measured with the Qubit® dsDNA BR Assay Kit (Thermo Fisher Scientific, Watham MA, USA), and DNA quality was assessed by the Fragment AnalyzerTM and the DNF-488 High Sensitivity genomic DNA Analysis Kit (Agilent, Santa Clare CA). DNA Methylation Profiling (RRBS Service) was performed by Diagenode (Diagenode Inc, Denville NJ, USA, #G02020000). Each experimental group for RRBS contained two biological replicates. RRBS libraries were prepared using the Premium Reduced Representation Bisulfite Sequencing (RRBS) Kit (Diagenode, #C02030033), with 100 ng of genomic DNA per sample used to start library preparation. PCR clean-up after the final library amplification was performed using a 1x (1.45 for second run) beads:sample ratio of Agencourt® AMPure® XP (Beckman Coulter, Brea, CA, USA). The quality of the RBBS library pools was evaluated by measuring their DNA concentration with the Qubit® dsDNA HS Assay Kit (Thermo Fisher Scientific, Watham MA, USA), and the profile of the pools was verified using the High Sensitivity DNA chip for 2100 Bioanalyzer (Agilent) or DNF-474 NGS fragment kit on a Fragment Analyzer (Agilent). Deep sequencing of RRBS library pools was performed with a NovaSeq6000 (Illumina) using 50 bp paired end read sequencing (PE50).

RRBS data processing was completed by Diagenode. Briefly, Bismark was used to align reads to a murine reference genome (mm10/GRCm38). Bisulfite conversion rates and efficiency were verified through the use of spike-in control sequences. Differential methylation analysis was conducted using Methylkit, an R/Bioconductor package. Pairwise comparisons were conducted to identify differentially methylated regions (DMRs), consisting of 1000 bp regions. To correct for multiple comparisons, *p* values were converted to q-values using the sliding window model (SLIM). Pairwise comparisons were conducted for the following comparisons: old stroke vs. old control and young stroke vs. young control. Significant DMRs are defined as having a methylation difference greater than 25% and q-value < 0.01. For post-data processing, all data analysis was conducted in R. Gene name, genomic region, and location in relation to CpG islands were identified for each DMR using annotator, an R/Bioconductor package. DMRs were classified by their genomic location, resulting in promoter DMRs and non-promoter DMRs, comprised of gene body DMRs and other DMRs. Gene body was defined as the 5ʹUTRs, 3ʹUTRs, introns, and exons, while other was defined as 1–5 kb upstream of transcription start sites or a lack of genomic location information. The following R packages were used for data visualization: ggplot2, VennDiagram and DOSE. The R/Bioconductor package clusterProfiler was used to conduct gene over-representation analysis with significant DMRs to identify significant gene ontology (GO) terms. The Benjamini–Hochberg procedure was used for *p* value correction for GO analysis. Pearson correlation was conducted to determine whether the percent change in methylation for common DMRs was significantly correlated across experimental groups.

### RNA sequencing

Total RNA was extracted from brain microvessels using TRIzol/chloroform, with concentration, purity, and integrity measured using agarose gel electrophoresis and an Agilent Bioanalyzer RNA 6000 Kit. RNA samples with RNA integrity number (RIN) score ≥ 5.8 were used to prepare libraries. RNA-seq library construction was generated using the Illumina NovaSeq 600 platform (Novogene Corporation Inc, Sacramento, CA USA). Total RNA was processed for poly(A) enrichment, followed by enzymatic fragmentation, cDNA synthesis, and double-stranded cDNA purification. Each experimental group for RNA-seq consisted of three biological replicates.

Data processing of the raw sequencing library was as follows: reads were trimmed using Cutadapt v2.3, data quality was confirmed with FastQC (v0.11.8), reads were mapped to the reference genome GRCm38 (ENSEMBL) using STAR v2.7.3a, and genes were assigned count estimates with RSEM v1.3.2 [53, 54]. Alignment options followed ENCODE standards for RNA-seq [53]. To ensure usage of high-quality data for expression quantitation and differential expression, FastQC was used in an additional post-alignment step. After obtaining count estimates, all data analysis was conducted in R. Differentially expressed genes (DEGs) were identified using DESeq2, defining cutoffs as adjusted *p* < 0.05 and absolute value of log2 fold change (Log2FC) > 0.58. Log2FC shrinkage of the RNA-seq data was conducted using the Ashr method. Data was visualized using the R packages ggplot2, VennDiagram and DOSE. Enriched GO terms were identified by gene over-representation analysis with significant DEGs in the R/Bioconductor package clusterProfiler. For gene over-representation analysis, *p* values were adjusted with the Benjamini–Hochberg procedure. Pearson correlation was conducted to determine whether the Log2FC for common DEGs was significantly correlated across experimental groups.

### Statistics

Additional statistical analyses were conducted in GraphPad Prism. Unpaired, two-tailed Student’s t-test was used to determine differences across experimental groups. The relationships between transcriptome and methylome profile in young and aging poststroke mice as well between young and aging methylome profile were evaluated using the Pearson correlation coefficient. Data are represented as mean ± SEM, with statistical significance defined as *p* < 0.05.

## Results

**Fig. 1:**
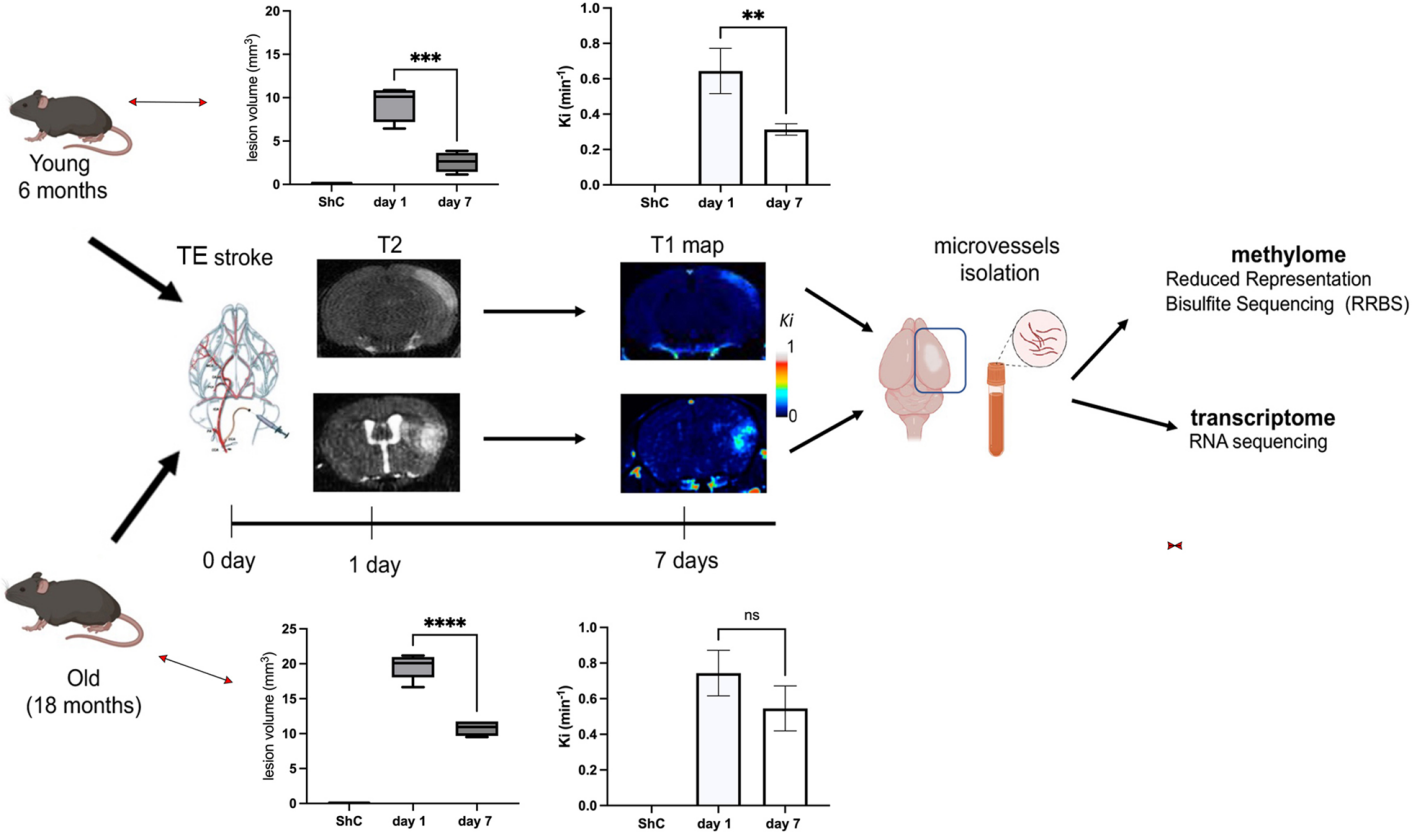
Experimental flow chart. Young and old mice underwent thromboembolic (TE) stroke. At one and seven days they underwent T2 MRI and T1 MRI to determine infarct size and BBB permeability (influx rate constant, Ki) before euthanasia and microvessel isolation, respectively. Those microvessels were used to determine the methylome and transcriptome changes. Graph represents means ± SD, *n* = 5, **p < 0.01, ***p < 0.001, ****p < 0.0001. Created with BioRender.com

Persistent BBB dysfunction with increased paracellular barrier permeability is present in chronic phases after stroke, questioning the recovery of the BBB (Fig. 1). To investigate the molecular mechanisms contributing to BBB recovery and detect potential causes of the limited BBB recovery, we profiled the DNA methylome and transcriptome of isolated microvessels from ischemic hemispheres 7 days after the induction of thromboembolic (TE) stroke via injection of thromboembolic suspension in two experimental groups: young mice aged 6 months and old mice aged 18 months. A diagram of the experimental flow is shown in Fig. 1.

### DNA methylome profile of the poststroke BBB recovery

**Fig. 2:**
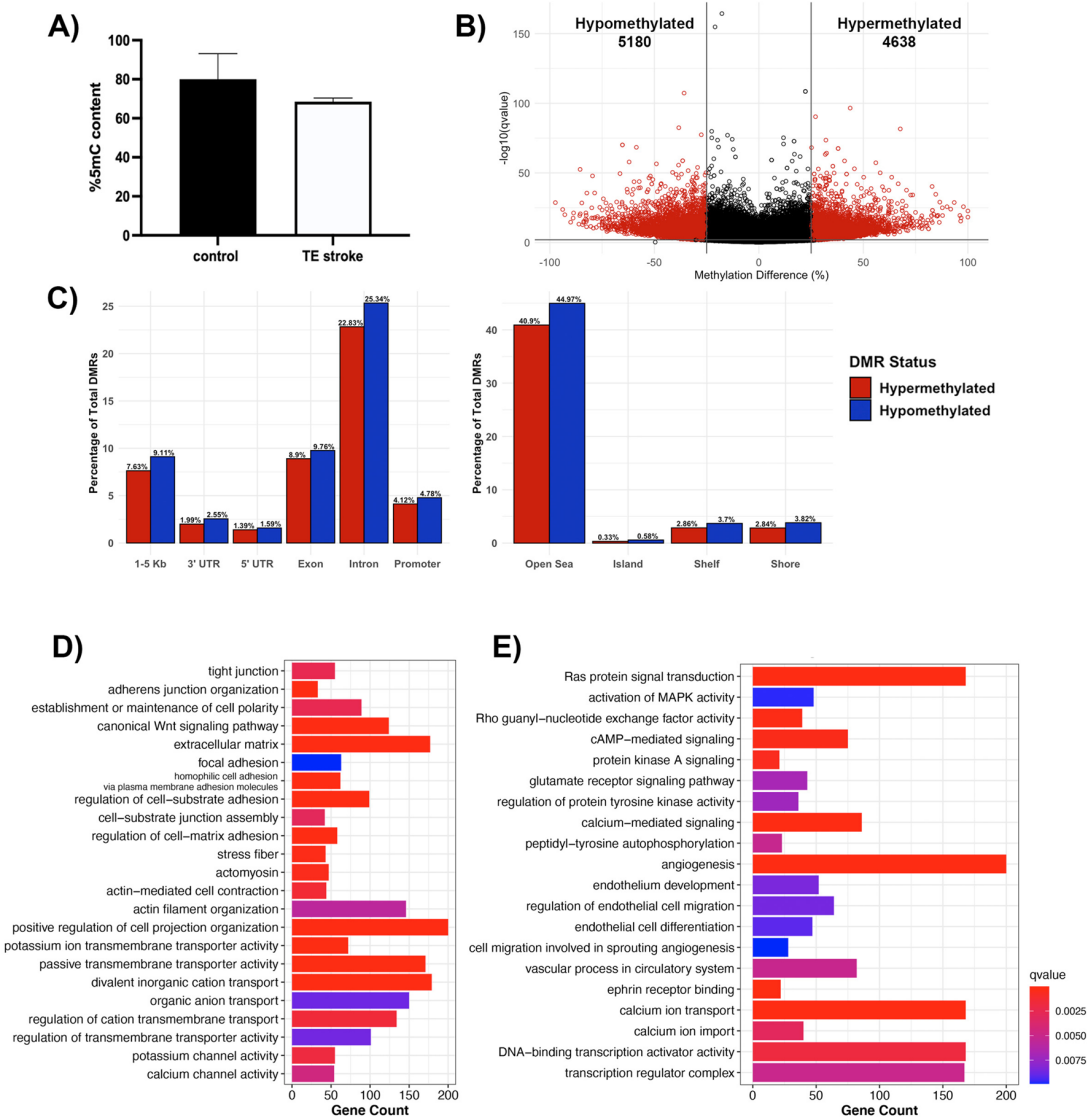
Analysis of DNA methylome in post-TE stroke BBB recovery. **A** Global DNA methylation assay demonstrates a trend of decreased global methylation following TE stroke in 6-month-old mice. Data represent average percentage of 5-mC ± SEM, n = 2. **B** Volcano plot of differentially methylated regions (DMRs), with the x- and y-axes showing percent methylation difference and -log10(q-value), respectively. Significant DMRs (methylation difference > 25% and q-value < 0.01) are represented in red, while DMRs with no statistical significance are black. **C** Bar charts summarizing the percentage of significant DMRs per genomic region (left) and CpG region (right). The percentage of hypermethylated DMRs are red, and the percentage of hypomethylated DMRs are blue. Summary of gene over-representation data for **D** structural GO terms and **E** signaling GO terms, with the x-axis demonstrating gene count. GO term were selected by statistical significance (q-value < 0.01) and relevance to endothelial cell biology

Global DNA methylation, determined by the percentage of 5-mC content, was not significantly altered following TE stroke in 6-month-old mice, although the TE stroke group showed slight hypomethylation comparing with sham control (control) group (Fig. 2a). As global methylation only captures large-scale changes to the DNA methylome, CpG-level changes were investigated through reduced representation bisulfite sequencing (RRBS), comparing the DNA methylome of brain microvessels from post-TE stroke mice to age-matched controls. During poststroke BBB recovery, we detected 9818 differentially methylated regions (DMRs, 1000 bp in size) with a percent change in methylation greater than 25% and a q-value < 0.01, with 4638 hypermethylated and 5180 hypomethylated DMRs (Fig. 2b). DMRs were primarily intronic (48.2%) and exonic (18.7%), while only 8.9% of DMRs were located in promoter regions (Fig. 2c). Strikingly, less than 1% of DMRs resided within CpG islands (Fig. 2c).

Categories of differentially methylated genes in post-stroke BBB recovery were identified through gene over-representation analysis. Overall, post-TE stroke changes to the DNA methylome largely affected genes encoding cell structural proteins (e.g., cell junction, and cell polarity, actin cytoskeleton, extracellular matrix, membrane microdomain), transporters and channels (e.g., potassium, organic anion and inorganic cation, calcium ion transport), and proteins involved in endothelial cell processes (e.g., angiogenesis and vasculogenesis, cell signaling and transcription regulation) (Fig. 2d, e).

**Fig. 3:**
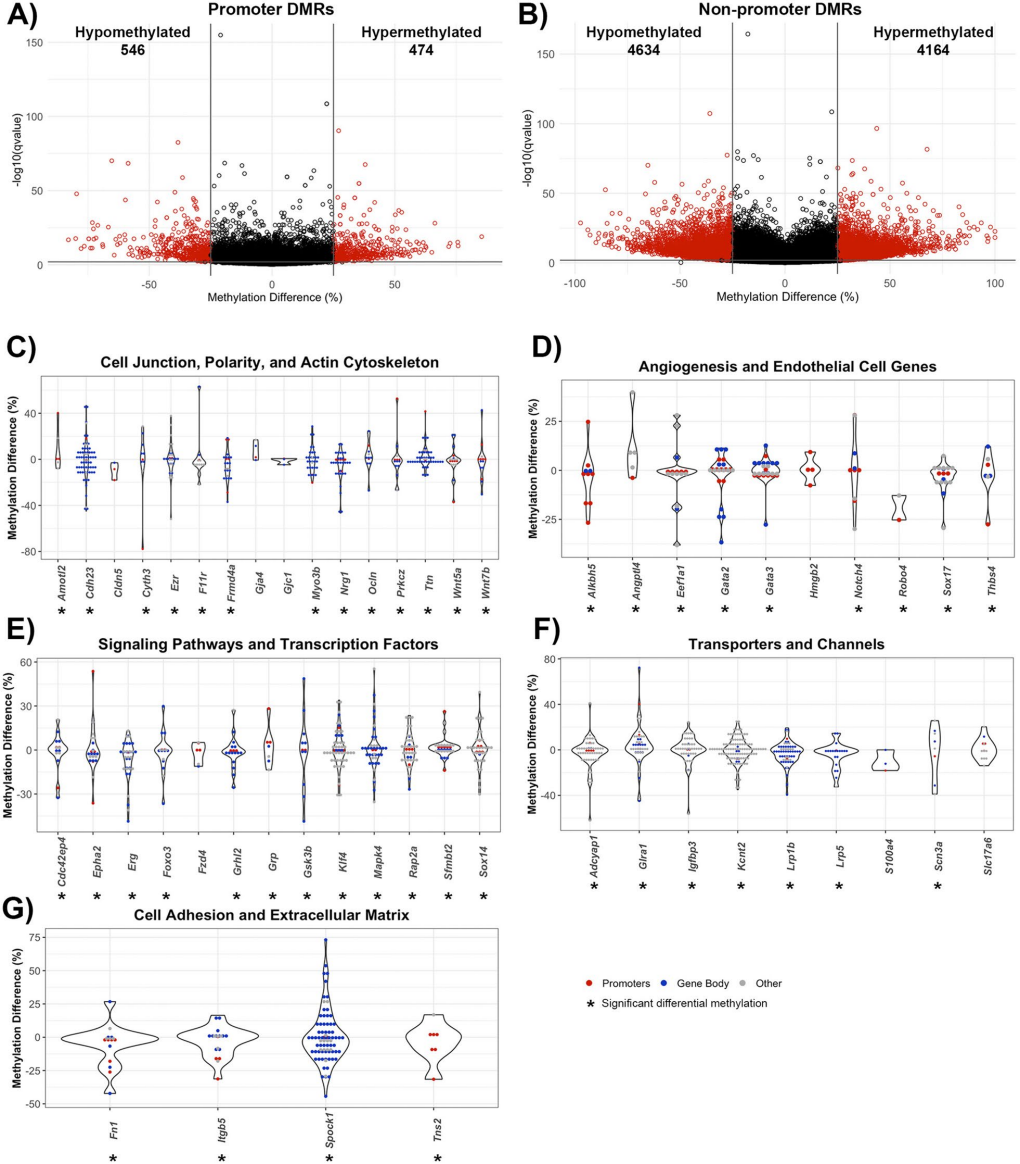
Genomic location of DMRs within poststroke BBB recovery in young (6 month) mice. Volcano plot of DMRs located within **A** gene promoters and **B** non-promoter regions, defined as any DMR outside of a promoter region (e.g. exons, introns, untranslated regions, 1-5 kb upstream of transcription start site). The x-axis is the percent methylation difference and the y-axis is the − log10(q-value). Significant DMRs (methylation difference > 25% and q-value < 0.01) are red, while DMRs lacking statistical significance are black. Violin plots demonstrate differential methylation of genes involved in (**C**) cell junctions, polarity and actin cytoskeleton, **D** angiogenesis and endothelial genes, **E** signaling pathways and transcription factors, **F** transporters and channels, and **G** cell adhesion and extracellular matrix. All DMRs, regardless of statistical significance, are represented for genes relevant to endothelial cell biology, with genes containing significant DMRs denoted with an asterisk (*). Gene promoter DMRs are red, and gene body DMRs are blue. Other DMRs, that are located 1-5 kb upstream of transcription start site or no genomic location, are gray

Due to their well-defined function in the context of transcription, DMRs within gene promoters and non-promoter regions were individually assessed, with non-promoter regions including the 5ʹ and 3ʹ untranslated regions (UTRs), introns, exons, and 1–5 kb upstream of transcription start sites. Out of the 1020 DMRs in gene promoters, 474 DMRs were hypermethylated and 546 DMRs were hypomethylated. (Fig. 3a). There were 8798 non-promoter DMRs, with 4164 DMRs hypermethylated and 4634 DMRs hypomethylated (Fig. 3b).

Prominent categories of differentially methylated genes in post-stroke BBB recovery were cell junction, polarity, and actin cytoskeleton. Significant cell junction GO terms included tight junction (q-value = 0.0025) and adherens junction organization (q-value = 0.00012). Notable differentially methylated genes with prevalent hypermethylation included junctional adhesion molecule-A (*F11r*), while prevalent hypomethylation occurred in occludin (*Ocln*) and claudin-5 (*Cldn5*) (Fig. 3c). Establishment or maintenance of cell polarity, a process required for proper localization of TJ complexes, was significant (q-value = 0.0025), with differential methylation observed in the Wnt signaling ligand genes, *Wnt5a* (hypomethylated mostly in promotor region) and *Wnt7b* (hypermethylated in gene body), the ARF6-regulating genes *Cyth3*, (hypomethylated in promotor region) and *Frmd4a* (hypermethylated in promoter and gene body), the endothelial polarity regulator *Amotl2*, as well as a regulator for establishing cell polarity *Prkcz,* (hypermethylation in promoter and gene body). Among the significant methylation changes in genes encoding actin cytoskeleton included those involved in actin filament organization (q-value = 0.0058) and positive regulation of cell projection organization (q-value = 2.85E−08), particularly within the actin-interacting genes *Ezr* and *Myo3b* (Fig. 3c).

In relation to poststroke BBB recovery, there were changes in a spectrum of genes associated with angiogenesis (q-value = 7.29E−06), endothelium development (q-value = 0.0084), regulation of endothelial cell migration (q-value = 0.0086) and endothelial cell differentiation (q-value = 0.0091). Significant differential methylation was present on the promoter and gene body of endothelial transcription factor genes include *Sox17*, *Gata2* and *Gata3* (all hypomethylated) while *Robo4*, involved in angiogenesis and endothelial barrier maintenance, had hypomethylated promoter DMRs. Strikingly, hypermethylation of the angiogenic inhibitor *Angptl4* was present but not *Hmgb2*, a promoter of endothelial cell proliferation and migration (Fig. 3d).

Altered methylation patterns were also found in genes encoding proteins involved in endothelial cell processes, such as intracellular signaling cascades and transcription (Fig. 2e). Examples include canonical Wnt signaling pathway (q-value = 8.74E−06), with differential methylation of the inhibitor *Gsk3b* but not the Wnt ligand receptor *Fzd4*, as well as Ras protein signal transduction (q-value = 1.74E−05), with differentially methylated (hypomethylated) genes including the GTPase *Cdc42ep4* and *Rap2a*, in both promotor and gene body DMRs. Other notable signaling cascade genes with hypomethylation include *Mapk4*, which regulates proinflammatory cytokines expression. Regarding transcriptional regulation, the DNA-binding transcription activator activity (q-value = 0.0018) and transcription regulator complex (q-value = 0.005) genes, such as *Foxo3* and *Grhl2,* were hypomethylated predominately in their gene bodies (Fig. 3e).

Transporter, receptor, and channel genes also had striking changes in methylation. For example, differentially methylated genes involved in passive transmembrane transporter activity (q-value = 0.00028) included the ion channel-encoding genes *Glra1*, *Kcnt2* and *Scn3a*. *Lrp1b* and *Lrp5*, low-density lipoprotein receptor family members, were hypomethylated predominantly in their gene bodies (Fig. 3f).

Extracellular matrix (ECM) genes (q-value = 6.74E−06) comprise the final category of differentially methylated genes, including genes involved in the regulation of cell-substrate adhesion (q-value = 1.28E−06), cell-substrate junction assembly (q-value = 0.003) and focal adhesion (q-value = 0.0099). Prominent promoter and gene body hypomethylation was present in *Fn1* (fibronectin), the fibronectin receptor *Itgb5,* and the focal adhesion molecule *Tns2* (Fig. 3g).

### Effect of methylome changes on the transcriptome profile of the poststroke BBB recovery

**Fig. 4:**
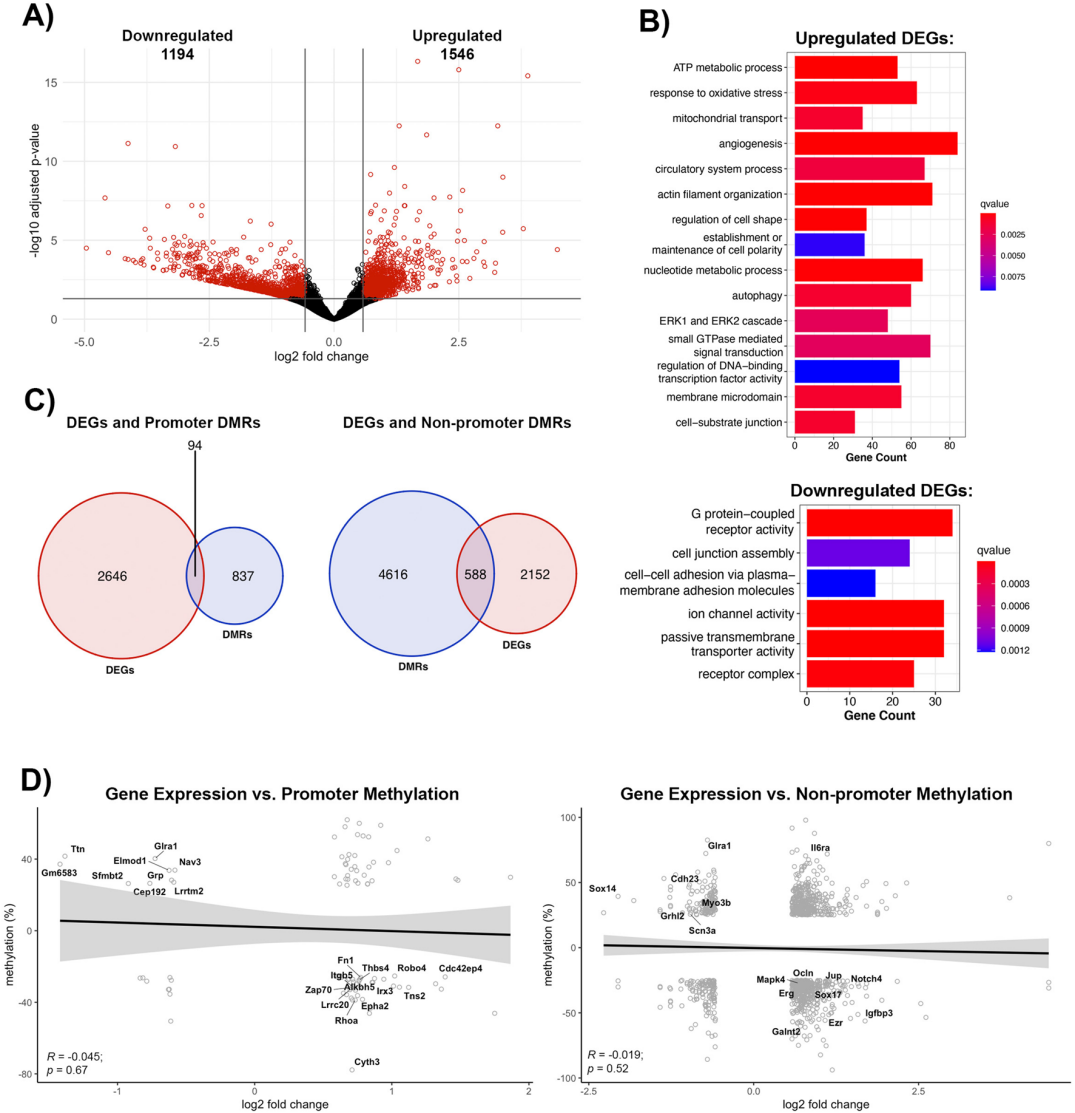
Comparison of poststroke BBB DNA methylome and transcriptome profile in young (6 month) mice. **A** Volcano plot of differentially expressed genes (DEGs). X-axis represents log2 fold change, and y-axis is the − log10(adjusted p-value). Significant DEGs (absolute value of log2FC > 0.58 and adjusted p-value < 0.05) are red, and DEGs with no statistical difference are black. **B** Gene over-representation analysis summaries for upregulated DEGs (top) and downregulated DEGs (bottom). Enriched GO terms were selected based on statistical significance (q-value < 0.01) and biological significance to endothelial cells. **C** Venn Diagrams demonstrating the number of DEGs with differentially methylated promoters (left) or non-promoter regions (right). Only significant DEGs and DMRs were counted. If a gene contained multiple DMRs, the gene was only counted once. **D** Pearson correlation between changes in gene expression and either promoter methylation (left) or non-promoter methylation (right) for genes with significant changes in gene expression and methylation, with log2FC on the x-axis and methylation changes on the y-axis. Neither promoter region methylation (R = − 0.045, p = 0.67) nor non-promoter region methylation (R = − 0.019, p = 0.52) negatively correlate with gene expression. Labeled genes are relevant to endothelial cell biology

The methylomic changes are best understood within the context of transcription, manifested as transcriptional repression due to promoter region methylation, while gene body methylation can lead to either transcriptional activation or repression [29, 30]. Thus, we performed a parallel analysis of transcriptomic changes in brain microvessels during post-stroke BBB recovery in young (6-months old) mice. Compared to age-matched controls, post-stroke BBB recovery resulted in 2740 differentially expressed genes (DEGs), defined as log2 fold change > 0.58 and an adjusted p-value < 0.05, with 1546 upregulated and 1194 downregulated (Fig. 4a). Gene ontology analysis revealed upregulated DEGs were involved in cell junction organization, such as cell-substrate junction (q-value = 0.001), establishment or maintenance of cell polarity (q-value = 0.009), ATP metabolic process (q-value = 5.66E−07), mitochondrial transport (q-value = 0.0010) and signaling cascades, such as ERK1 and ERK2 cascade (q-value = 0.0026) and small GTPase mediated signal transduction (q-value = 0.0028). Other enriched GO terms for upregulated DEGs included angiogenesis (q-value = 9.10E−06) and regulation of DNA-binding transcription factor activity (q-value = 0.0091). Downregulated DEGs demonstrated an enrichment of GO terms related to cell junctions, such as cell junction assembly (q-value = 0.0011) and cell–cell adhesion via plasma membrane adhesion molecules (q-value = 0.0012), ion channel activity (q-value = 1.63E−08), passive transmembrane transporter activity (q-value = 3.84E−08), and signaling pathways, such as G protein-coupled receptor activity (q-value = 1.62E−10) (Fig. 4b).

As promoter and gene body methylation directly affect the transcriptome, we assessed the overlap between changes to the DNA methylome and the transcriptome. Only 94 DEGs had altered promoter methylation, while 588 DEGs had differential methylation of non-promoter regions (Fig. 4c). However, the correlation between promoter methylation and gene expression demonstrated a non-significant trend toward a negative correlation (R = − 0.045, *p* = 0.67). Despite that, there are groups of genes with significant promoter hypomethylation (q-value) and transcript upregulation (p.adjust), like the RhoGTPases, *Cdc42ep4* (p.adjust = 0.0004, q-value = 7.94E−09) and *Rhoa* (p.adjust = 0.0236, q-value = 4.28E−12), focal adhesion molecule *Tns2 *(p.adjust = 0.00255, q-value = 3.74E−20), the regulator of angiogenesis and endothelial barrier establishment *Robo4* (p.adjust = 0.0051, q-value = 3.15E−09*),* integrin β5 (*Itgb5,* p.adjust = 0.032, q-value = 0.00014), extracellular matrix protein fibronectin (*Fn1,* p.adjust = 0.032, q-value = 3.34E−29) and the angiogenic factors thrombospondin 4 (*Thbs4*, p.adjust = 0.04, q-value = 2.14E−17) and *Alkbh5* (p.adjust = 6.56E−05, q-value = 1.66E−13). Promoter hypermethylation and transcript downregulation was observed in the regulator of VEGF-induced angiogenesis and glycine-mediated vascular reconstruction *Glra1* (p.adjust = 0.043*,* q-value = 3.29E−05), the transcriptional repressor Scm-like with four MBT domains protein 2 (*Sfmbt2,* p.adjust = 0.017, q-value = 7.69E−08), the regulator of mechanotransduction *Ttn* (p.adjust = 0.001, q-value = 1.28E−26), cell polarity protein *Cyth3* (p.adjust = 0.0124, q-value = 1.16E−11) and *Epha2* (p.adjust = 0.031, q-value = 2.19E−05) (Fig. 4d, left).

Similar to promoter changes, methylation in non-promoter regions showed a non-significant negative correlation with transcriptome expression (R = − 0.019, *p* = 0.52), although a positive correlation exists between gene expression and non-promoter methylation for a group of genes. Genes with hypomethylated non-promoter regions associated with significant transcript upregulation include the TJ protein occludin (*Ocln*, p.adjust = 0.032, q-value = 1.11E−07) adherens junction protein plakoglobin (*Jup*, p.adjust = 0.00068, q-value = 2.9E−13), actin cytoskeleton linker protein ezrin (*Ezr*, p.adjust = 0.0056, q-value = 2.28E−10), the regulator of the Wnt signaling and BBB maintenance *Sox17* (p.adjust = 0.01, q-value = 1.80E−07), the angiogenic transcription factor *Erg* (p.adjust = 0.038, q-value = 2.94E−37), along with signaling molecules that promotes angiogenesis and barrier permeability, such as *Mapk4* (p.adjust = 1.15E−05, q-value = 1.03E−07), and *Igfbp3* (p.adjust = 0.00024, q-value = 7.72E−13), and the angiogenic inhibitor *Notch4* (p.adjust = 0.00016, q-value = 2.46E−05). Furthermore, hypermethylation of non-promoter regions is associated with downregulation of genes encoding cell adhesion and mechanotransduction *Cdh23* (p.adjust = 9.57E−07, q-value = 1.63E−15), actin cytoskeleton and mechanotransduction myosin 3b (*Myo3b,* p.adjust = 0.037, q-value = 1.37E−05), the transcription factor and regulator of Wnt/b-catenin signaling pathways *Sox14* (p.adjust = 0.0032, q-value = 0.0002), the potassium transporter *Scn3a* (p.adjust = 0.0084, q-value = 1.17E−07) and the regulator of cell projection and morphogenesis *Grhl2* (p.adjust = 0.036, q-value = 8.72E−16; Fig. 4d, right). Taken together, the methylome and transcriptome profile of BBB recovery indicated extensive remodeling of barrier properties mirrored by structural alterations (TJ protein expression, actin cytoskeleton remodeling, reestablishing cell polarity), and a restoration of the extracellular matrix and transporter systems. The brain endothelial cells display a more proangiogenic phenotype with activation of angiogenic transcription factors and Wnt-β-catenin signaling pathways for remodeling barrier properties. In addition to Wnt-β-catenin, other prominent signaling pathways that can alter recovery outcomes include Rho GTPase and MAPK.

### Effect of aging on the BBB DNA methylome and transcriptome profile in poststroke recovery

**Fig. 5:**
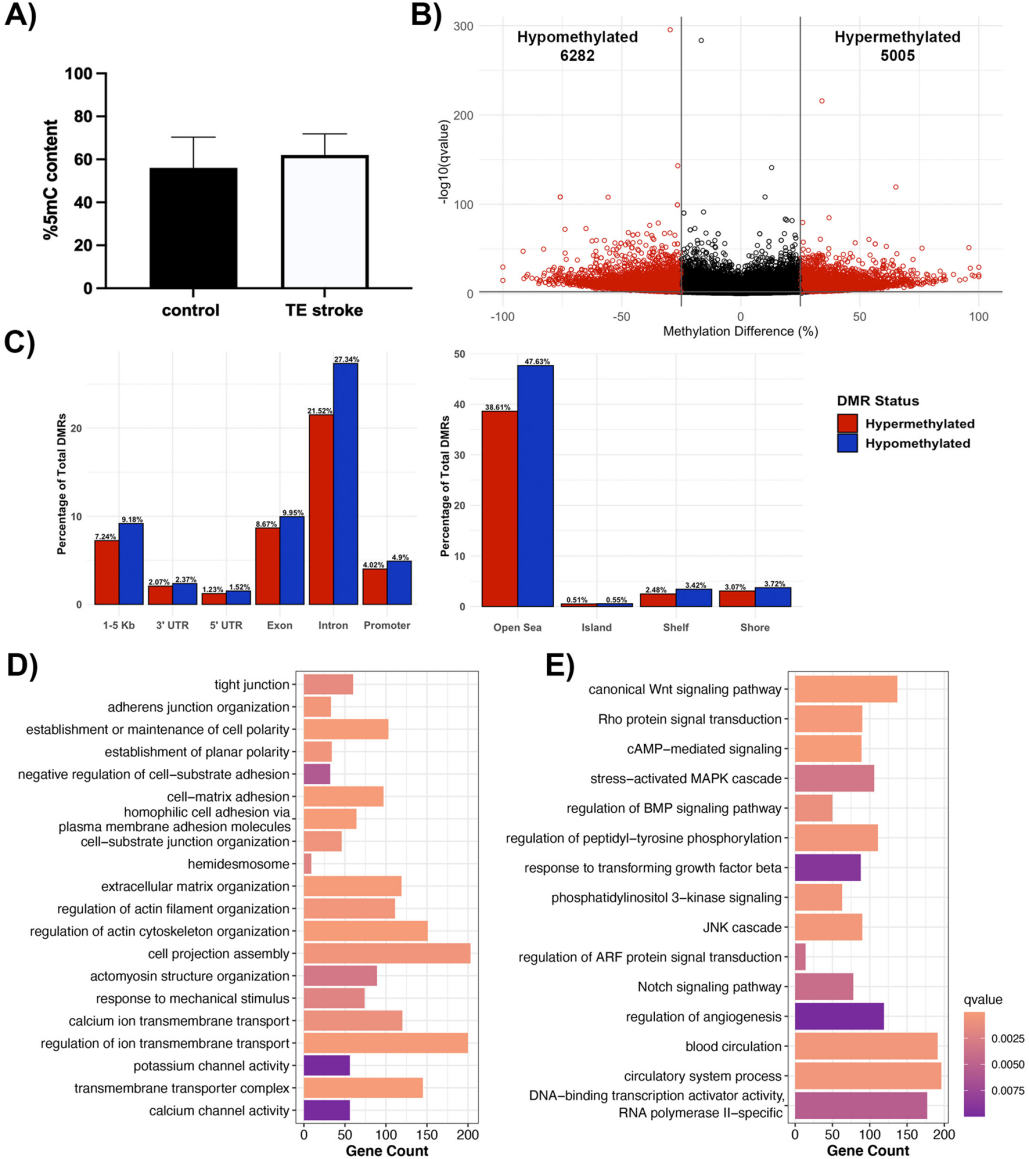
Effect of aging on the DNA methylome profile in poststroke BBB recovery **A** Global DNA methylation assay demonstrates no change in global methylation in isolated microvessels (BBB) post-TE stroke in aging mice (18 months). Data represents the average percentage of 5-mC content ± SEM, n = 2. **B** Volcano plot of DMRs in old post-TE stroke mice, with percent methylation difference on the x-axis and − log10(q-value) on the y-axis. Significant DMRs are highlighted in red (methylation difference > 25% and q-value < 0.01), while nonsignificant DMRs are black. **C** Bar charts demonstrating the percentage of significant DMRs per genomic region and CpG region. Hypermethylated DMRs are represented in red and hypomethylated DMRs in blue. Summary of enriched **D** structural and **E** signaling GO terms from gene over-representation analysis. GO terms were selected based on their biological and statistical significance (q-value < 0.01)

Aging plays a critical role in the epigenetic alteration of the brain endothelial cells function and consequently on barrier properties [55]. Aged mice (18 months) had larger infarct sizes with profound BBB leakiness 7 days after TE stroke onset (Fig. 1). Analyzing DNA methylome profile in post-stroke BBB recovery in aged mice, we found no changes in the global methylation level (Fig. 5a). However, RRBS analysis revealed that aging post-TE stroke brain microvessels had 11,287 DMRs, 5005 hypermethylated and 6282 hypomethylated, compared to age matched controls (Fig. 5b). The genomic regions containing the highest percentages of DMRs were introns, exons, and 1–5 kb upstream of the transcriptional start site, respectively while only 8.9% of DMRs were located within promoter regions (4.0% hypermethylated and 4.9% hypomethylated). When investigating DMR location in relationship to CpG islands, most (86.2%) were located within the open sea, while only 1.1% of DMRs were located within CpG islands (Fig. 3c).

**Fig. 6:**
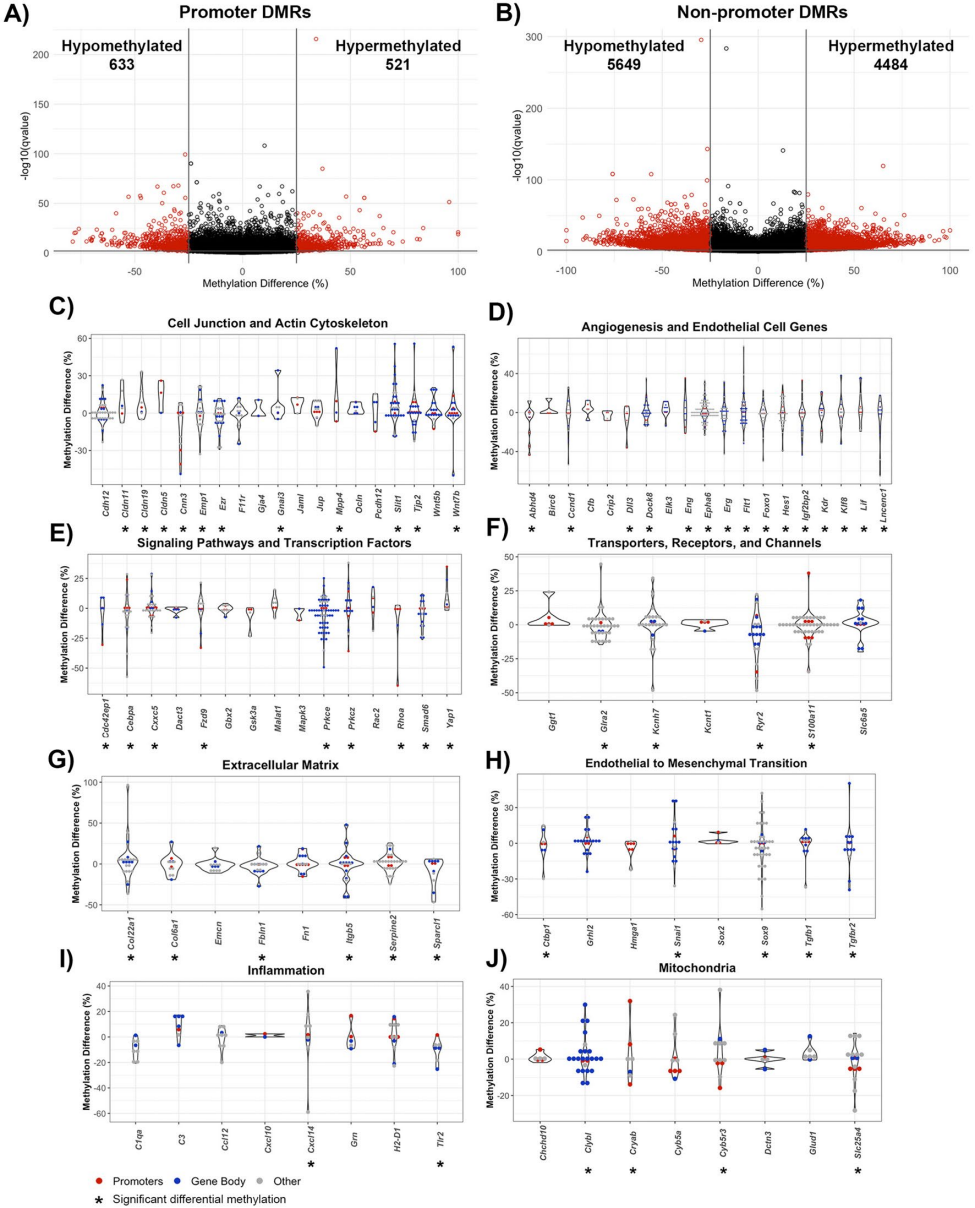
Genomic location of DMRs in “aged” poststroke BBB. Volcano plots of old post-stroke DMRs within **A** gene promoters **B** non-promoter regions. The x-axis represents percent methylation difference from age-matched controls and the y-axis reflects − log10(q-value). Significant DMRs are depicted in red (methylation differences > 25% and q-value < 0.01), and nonsignificant DMRs are in black. Violin plots of DMRs located within genes involved in **C** cell junction and the actin cytoskeleton, **D** angiogenesis and endothelial genes, **E** signaling pathways and transcription factors, **F** transporters, receptors, and channels, **G** extracellular matrix, **H** endothelial to mesenchymal transition, **(I)** inflammation, **J** mitochondria. All DMRs, regardless of significance, are plotted for select endothelial genes. Genes containing significant DMRs are marked with an asterisk (*). The y-axis represents percent methylation difference, with DMRs colored by genomic location: promoters in red, gene body in blue, and other (1–5 kb upstream of transcription start site or no genomic location) in gray

Similar to poststroke BBB recovery in young mice, poststroke BBB in aging mice had gene enrichment in clusters of cell junctions, actin cytoskeleton, angiogenesis, signaling pathways and transcription factors, transporters, and channels, as well as the extracellular matrix (Fig. 5d, e). The promoter regions showed 1154 DMRs (521 hypermethylated and 633 hypomethylated) (Fig. 6a), while non-promoter regions had 10,133 DMRs, 4484 being hypermethylated and 5649 being hypomethylated (Fig. 6b). The significant methylation pattern in promoter and non-promoter regions were present in the tight junction cluster (q-value = 0.0015), with hypermethylation of genes that encode *Cldn5*, *Tjp2*, and *Ocln* and hypomethylation of *F11r*, actin cytoskeleton (e.g. regulation of actin filament organization, q-value = 0.0004; cell projection assembly, q-value = 0.0005; and actomyosin structure organization, q-value = 0.0031) with notable hypomethylation of actin cytoskeleton-related genes that encode *Ezr*, and filament-associated protein *Cnn3*. Two genes that regulate the BBB recovery and maintenance, *Wnt5b* and *Wnt7a*, showed hypermethylation and hypomethylation, respectively, predominantly in gene body (Figs. 5c, 6c).

Another cluster with significantly altered methylation related to angiogenesis and endothelial cell function. That included regulation of angiogenesis (q-value = 0.00999), blood circulation (q-value = 0.00044), and circulatory system processes (q-value = 0.00041). Hypomethylation was detected in genes encoding VEGF receptors, *Flt1* and *Kdr*, and transcription factors involved in endothelial cell function (e.g., *Foxo1, Hes1, Klf8* and *Erg*), while other essential endothelial cell transcription factors showed more hypermethylated pattern (e.g., *Crip2* and *Elk3*) (Fig. 6d). In the cluster of signaling pathways and transcription factors, significant hypomethylation occurred in genes encoding canonical Wnt signaling pathway (q-value = 3.35E−07) with *Fzd9* having a hypomethylated promoter, RhoGTPAse (q-value = 0.00016) with hypomethylated promoter DMRs within *Cdc42ep1* and *Rhoa*, and Protein kinase C encoding genes (belonging to regulation of peptidyl-tyrosine phosphorylation, q-value = 0.00047) including *Prkce* and *Prkcz*. Other notable changes were present in the regulation of BMP signaling pathway (q-value = 0.0011) and response to transforming growth factor beta (q-value = 0.0091), with *Smad6* containing DMRs (Fig. 6e). Differentially methylated genes encoding transporters, channels, and receptors were calcium ion transmembrane transport (q-value = 0.00098) and calcium channel activity (q-value = 0.009996), with DMRs located within *Ryr2*, potassium channel activity (q-value = 0.009996), the voltage-gated potassium channel subunit *Kcnh7m* and the calcium-binding protein *S100a11* (Fig. 6f). Another significant category was regulation of ion transmembrane transport (q-value = 7.14E−05) and transmembrane transporter complex (q-value = 3.59E−07) (Fig. 6f). The extracellular matrix cluster was also significantly enriched with genes that regulate cell–matrix adhesion (q-value = 3.08E−05), cell-substrate junction organization (q-value = 0.00068), negative regulation of cell-substrate adhesion (q-value = 0.0059), and extracellular matrix organization (q-value = 0.00010). Genes of interest with altered methylation include *Fn1*, which encodes fibronectin (hypermethylation), along with its binding partner *Fbln1*, *Coll6a1, Sparcl1* and integrin-β5 (*Itgb5*) (Fig. 6g).

Unique to old post-stroke BBB recovery included enrichment of DMRs in genes involved in endothelial to mesenchymal transition (EndMT), inflammation, and mitochondria function (Fig. 5c, d). Interestingly, EndMT genes with differential methylation were *Tgfb1* and its receptor, *Tgfbr2*, transcription factors such as *Ctbp1* and *Snai1* as well *Sox9, Sox2*, another SRY-related HMG-box family member, *Grhl2* and *Hmga1* (Fig. 6h). Among inflammatory mediators, prominent DMRs were located within genes of the complement system (e.g., *C1qa* and *C3*), various cytokines and chemokines (e.g*., Cxcl14, Ccl12* and *Cxcl10*), toll like receptor 2 (*Tlr2)*, lysosomal function (e.g., *Grn*), and histocompatibility complexes (e.g., *H2-D1*; Fig. 6i). Mitochondrial-associated genes also demonstrate altered methylation patterns; for example, the ADP:ATP antiporter, *Slc25a4* (hypomethylation), cytochrome-encoding gene *Cyb5r3*, and metabolic enzyme *Clybl* and *Glud1* (Fig. 6j). Nevertheless, altered methylation with similar direction and trend was present in the non-stroke aging groups implying that aging process could be a determinant of poststroke recovery process in BBB. For example, EndMT transcription factor *Sox9* (q-value = 7.61E−09) and *Snai1* (q-value = 1.81E−07), chemokine *Ccl10* (q-value = 2.30E−06), proinflammatory MAP kinase Mapk4 (q = 1.44E−19) were hypomethylated while TJ occlusion protein *Cldn5* (q-value = 1.13E−33) and cytochrome-encoding gene *Cyb5r3* (q-value = 6.34E−17) were hypermethylated in gene promotor region in aging control (non-stroke) group compared to the young control group (data not shown).

**Fig. 7:**
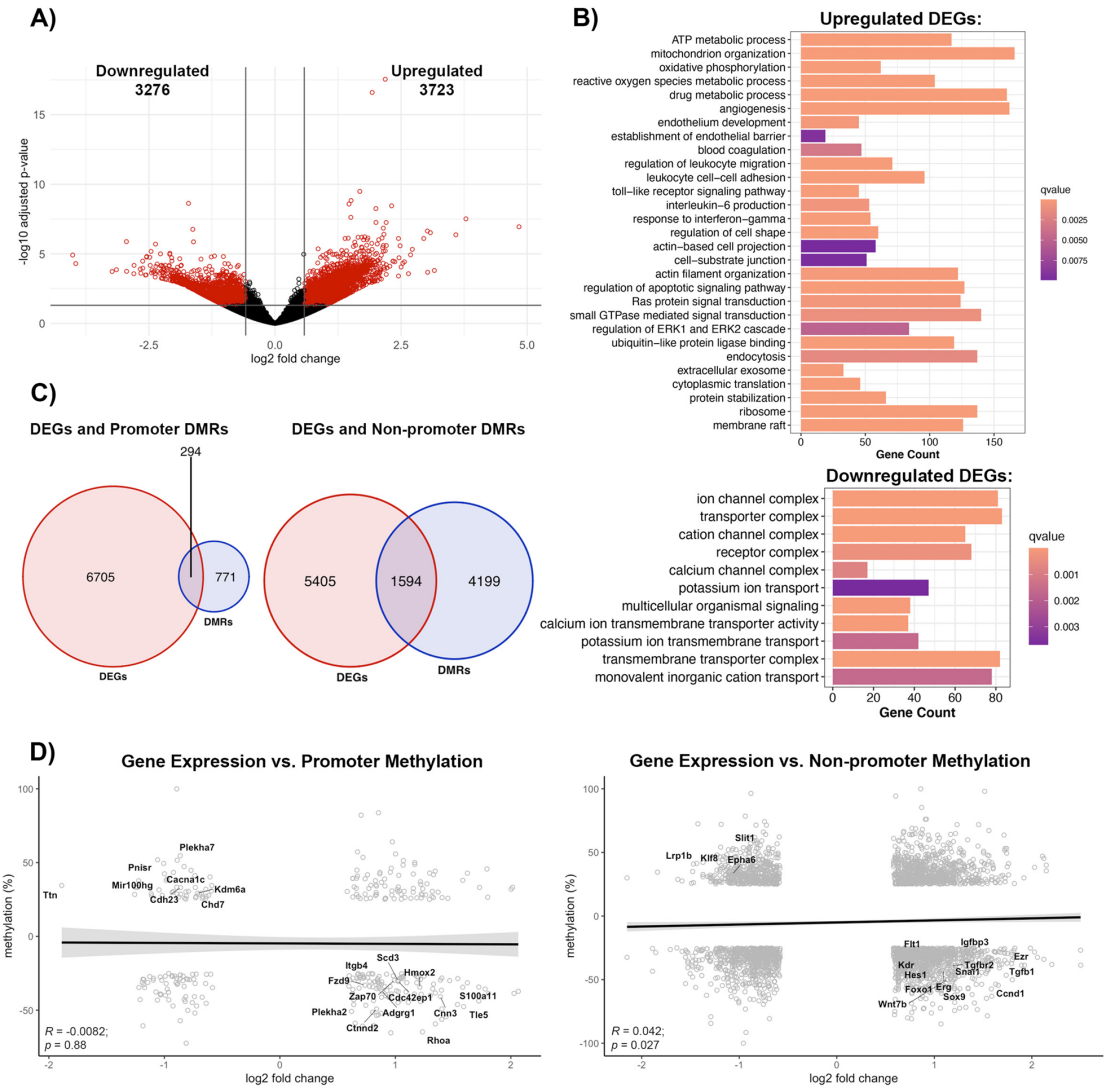
Comparison of poststroke BBB DNA methylome profile in aging mice **A** Volcano plot of DEGs in aged BBB post-TE stroke condition, with log2 fold change on the x-axis and − log10(adjusted p-value) on the y-axis. Significant DEGs are red (absolute value of log2 fold change > 0.58 and adjusted p-value < 0.05), while DEGs with no statistical significance are black. **B** Summary of gene over-representation analysis for upregulated DEGs (top) and downregulated (bottom) DEGs from gene over-representation analysis. GO terms were chosen based on statistical (q-value < 0.01) and biological significance. **C** Venn diagrams demonstrating the number of DEGs with altered promoter methylation (right) or non-promoter methylation (left). **D** Pearson correlation between changes in gene expression and promoter methylation (left) or non-promoter methylation (right) for genes with significant changes in expression and methylation. DEGs do not correlate with methylation of their promoters (R = -0.0082, p = 0.88); however, DEGs positively correlate with methylation of non-promoter regions (R = 0.042, p = 0.027). Labeled genes are biologically significant

The altered methylation pattern was analyzed in the context of transcriptomic changes. RNA-seq revealed that poststroke BBB recovery in old mice have global transcriptomic changes compared to age-matched controls: out of the 6999 total DEGs, 3723 were upregulated, and 3276 were downregulated (Fig. 7a). Most of the upregulated genes were enriched in the following categories: angiogenesis (q-value = 7.69E−08), cell-substrate junction (q-value = 0.0097), actin filament organization (q-value = 0.00014), regulation of apoptotic signaling pathway (q-value = 3.87E−0), Ras protein signal transduction (q-value = 0.00046), regulation of ERK1 and ERK2 cascade (q-value = 0.0050), inflammatory processes, such as regulation of leukocyte migration (q-value = 2.57E−06), and metabolism, including ATP metabolic processes (q-value = 9.08E−20) and oxidative phosphorylation (q-value = 1.98E−17) (Fig. 7b). Intriguingly, upregulated DEGs were enriched for GO terms relating to translational control, which was unique to the old poststroke group. This included cytoplasmic translation (q-value = 8.02E−10) and ribosome (q-value = 1.04E−37) (Fig. 7b). Furthermore, enriched GO terms for downregulated DEGs included calcium ion transmembrane transporter activity (q-value = 2.79E−06), potassium ion transport (q-value = 0.0037), calcium channel complex (q-value = 0.0008), and transmembrane transporter complex (q-value = 1.32E−14) (Fig. 7b).

Comparing the overlap between DEGs and DMRs revealed only 294 DEGs with altered methylation within their promoter regions, and 1594 DEGs with altered methylation within non-promoter regions (Fig. 7c). Similar to the poststroke condition in young mice, expression of select DEGs negatively correlated with their promoter region methylation (R = − 0.0082, *p* = 0.88); however, methylation of non-promoter regions positively and significantly correlated with gene expression (R = 0.042, *p* = 0.027). (Fig. 7d). Despite this positive correlation, there are groups of genes that have a negative correlation between their expression and methylation pattern. Hypomethylated genes with upregulated transcript expression included the actin cytoskeleton protein ezrin (*Ezr*, p.adjust = 6.75E−05, q-value = 8.26E−10) and actin-related protein *Cnn3* (p.adjust = 0.00028, q-value = 2.68E−10), regulators of angiogenesis like Vegf receptors *Flt1* (p.adjust = 0.031, q-value = 1.76E−14) and *Kdr* (p.adjust = 0.0232, q-value = 5.68E−07), angiogenic transcription factors *Erg* (p.adjust = 0.0117, q-value = 2.81E−06) and *Hes1* (p.adjust = 0.0013, q-value = 2.48E−06) and the cell cycle regulatory axis *Ccnd1* (p.adjust = 3.24E−05, q-value = 1.22E−06)/*Igfbp3* (p.adjust = 0.0008, q-value = 3.83E−35). Members of the Wnt canonical pathway and regulators of the BBB repair, *Wnt7b* and *Fzd9*, were hypomethylated (Wnt7b q-value = 8.83E−13, and Fzd9 q-value = 1.47E−07) with significant upregulation of gene expression (Wnt7b p.adjust = 0.0022; Fzd9, p.adjust = 0.0188). Besides Wnt signaling pathways, promoter hypomethylation and transcript upregulation was observed for *Rhoa* (p.adjust = 0.0008, q-value = 3.68E−14) and *Cdc42ep1* (p.adjust = 0.0014, q-value = 5.47E−05) (Fig. 7d).

Intriguingly, old poststroke BBB recovery also resulted in the significant hypomethylation and subsequent upregulation of a repressor of angiogenesis, the transcriptional factor *Foxo1* (p.adjust = 0.0034, q-value = 2.37E−06) and hypermethylation/downregulation of two angiogenic factor genes, the transcription factor *Klf8* (p.adjust = 0.0011, q-value = 8.31E−28) and *Epha6* (p.adjust = 0.0010, q-value = 6.13E−09) (Fig. 7d). Ultimately, altered methylation and expression of these genes could affect the outcome of poststroke angiogenesis. Regarding structural proteins responsible for building the barrier, hypermethylation of a non-promoter region in *Slit1* is associated with its decreased transcript expression (p.adjust = 0.0483, q-value = 1.27E−14), while promoter hypermethylation of *Cldn5* was associated with upregulated transcript expression (p.adjust = 0.0055, q-value = 2.66E−09) (Figs. 6b, 7d). For old poststroke mice, a unique methylation pattern exists in genes encoding endothelial to mesenchymal transformation. Hypomethylation within non-promoter regions and increased transcript expression is present for genes that encode *Snai1* (p.adjust = 0.0086; q-value = 2.84E−07), *Sox9* (p.adjust = 0.0006, q-value = 7.76E−09), *Tgfb1* (p.adjust = 6.90E−05, q-value = 8.91E−63) and *Tgfbr2* (p.adjust = 0.0047, q-value = 1.80E−09).

In summary, the transcriptional and methylome profile of poststroke BBB recovery in aging mice implies that angiogenesis and Wnt-related pathways drive the BBB recovery process, although the repair process is affected by the activation of genes involved in endothelial to mesenchymal transformation and angiogenic repression, limiting the final outcomes.

### Common and unique transcriptomic and DNA methylome profiles of BBB poststroke recovery in young and aging mice

**Fig. 8:**
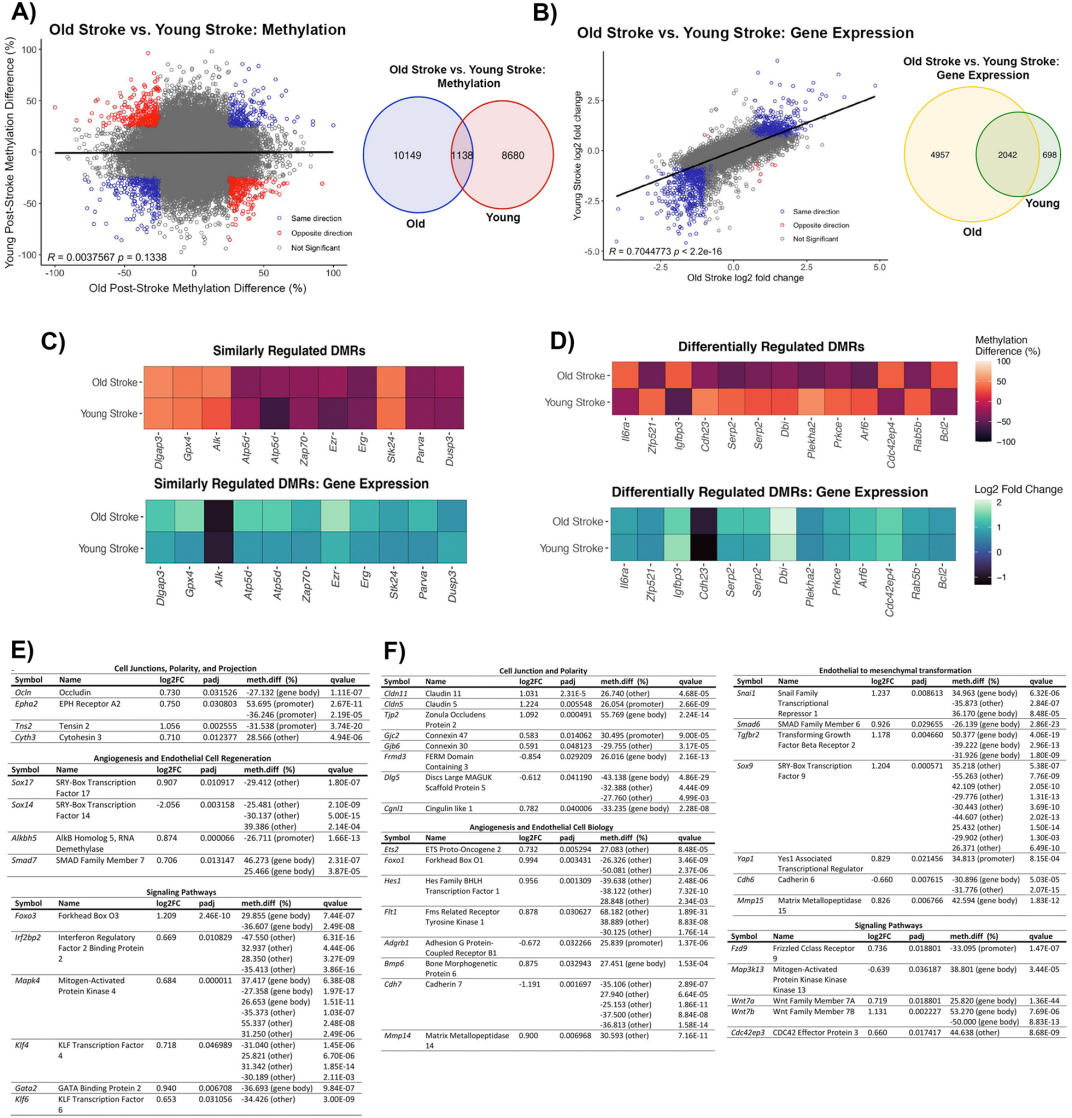
Common and unique DEGs with altered DNA methylation in poststroke BBB in young and old mice **A** Pearson correlation (left) between DMRs common to poststroke BBB in old (18 months) and young (6 months) mice, regardless of genomic location, with percent methylation difference of old post-TE stroke and young post-TE stroke on the x- and y-axes, respectively. Statistically significant DMRs with methylation changes in the same direction (e.g., hypermethylated in both groups) are blue, statistically significant DMRs with methylation changes in the opposite direction (e.g., hypermethylated in aging post-TE stroke and hypomethylated in young post-TE stroke) are red. Venn diagram (right) demonstrates the number of common DMRs between the old and the young post-TE stroke mice. Only statistically significant DMRs are represented, with direction of change (i.e., hypermethylation or hypomethylation) not considered. **B** Pearson correlation (left) between common DEGs for old and young post-TE stroke mice, with log2 fold change of DEGs in old post-TE stroke mice and young post-TE stroke mice on the x- and y-axes, respectively. Statistically significant DEGs regulated in the same direction (e.g., upregulated transcript expression in both groups) are blue, and statistically significant DEGs regulated in opposite directions (e.g., upregulated in aging post-TE stroke mice and downregulated in young post-TE stroke mice) are red. Venn diagram (right) showing the number of common DEGs between old post-stroke TE mice and young post-TE stroke mice. Data reflects statistically significant DEGs, with direction of change, i.e., upregulation or downregulation, not considered. **C** Heatmaps demonstrating examples of genes with DMRs regulated similarly across old post-TE stroke and young post-TE stroke (top) and their changes in gene expression (bottom). **D** Heatmaps depicting examples of genes with DMRs regulated differentially between the two experimental groups (top) and their changes in gene expression (bottom). **E** Tables demonstrating DEGs with altered methylation unique to poststroke BBB of young (6 months) mice. Genes are categorized based on their cellular process. **F** Tables demonstrating DEGs with altered methylation unique to poststroke BBB in old (18 months) mice, with genes classified by their cellular process. Genes were selected based on their statistical significance and their importance in endothelial cell function

As the severity of poststroke BBB injury differs between young and old mice, we compared the DNA methylome and transcriptome changes to highlight cellular processes contributing to the discrepancy in poststroke BBB recovery. Remarkably, there are only 1138 significant DMRs common to poststroke BBB recovery in both old and young mice (Fig. 8a). This lack of overlap is still observed when assessing methylation within specific genomic regions, as there are 112 common DMRs within promoters and 1026 DMRs within non-promoter regions (Additional file 1: Fig. S1a, b). Common DMRs identified in poststroke BBB recovery of old and young mice do not significantly correlate (R = 0.0038, *p* = 0.134), which is also true when assessing the correlation of common non-promoter DMRs (R = 0.0018, *p* = 0.5227) (Fig. 8a, Additional file 1: Fig. S1d). A significant correlation, however, is observed for the common promoter region DMRs (R = 0.0188, p-value = 0.00055) (Additional file 1: Fig. S1c). Common DMRs with methylation changes in the same direction (e.g., hypermethylated in both groups) belong to signaling pathways (e.g., positive regulation of MAPK cascade, q-value = 0.03; GTPase regulator activity, q-value = 0.015; and G protein-coupled receptor activity, q-value = 0.041) (Additional file 1: Fig. S1e). DMRs with methylation changes in opposite directions (e.g., hypermethylated in old and hypomethylated in young) are located within genes involved in endocytosis (e.g., clathrin-coated endocytic vesicle, q-value = 0.0187; endocytic vesicle, q-value = 0.039) and b-catenin binding (q-value = 0.038) (Additional file 1: Fig. S1f). Comparing the transcriptomic changes in BBB recovery across the young and old mice reveals 2042 significant DEGs common to both experimental groups (Fig. 8b). The common DEGs between old and young poststroke BBB recovery have a strong positive correlation (R = 0.704, *p* < 2.2E−16), with only 8 DEGs regulated in the opposite direction (e.g., upregulated in old post-TE stroke mice and downregulated in young post-TE stroke mice) (Fig. 8b).

Furthermore, we identified genes of interest with similar changes in methylation (e.g., hypermethylated or hypomethylated in both experimental groups) for both young and old mice. These genes encode proteins involved in actin-binding signaling pathway activity, transcriptional regulation, and protection from oxidative stress. For example, actin-binding proteins *Parva* and *Ezrin*, and the Ets family transcription factor *Erg* were hypomethylated with increased transcript expression in the poststroke microvessels of both young and old mice. Other examples included the activator of MAPK signaling *Alk* (hypermethylated and decreased transcript expression) and the negative regulator of MAPK signaling *Dusp3* (hypomethylated and increased transcript expression), indicating the same trend in the regulation of MAPK kinase in BBB recovery (Fig. 8c). Genes with differential methylation patterns (e.g., hypermethylated in old and hypomethylated in young) across the BBB of old and young poststroke mice include the signaling molecule *Prkce*, involved in actin cytoskeleton function (migration, adhesion) and actin cytoskeleton modulator *Arf6* (both genes hypomethylated in old mice and hypermethylated in young mice), while *Cdc42ep4* was hypermethylated in old and hypomethylated in the young post-stroke BBB. Conversely, increased transcript expression of *Arf6* and *Cdc42ep4* was observed in old and young post-stroke BBB (Fig. 8c).

Finally, there is a unique DNA methylome and transcriptome profile for both experimental groups. These genes belong to categories relevant to endothelial cell biology, such as cell junctions, angiogenesis, and signaling pathways. The unique pattern for poststroke BBB recovery in young mice was characterized by alterations in methylation and transcriptome expression of cell junction and polarity complex regulators such as *Ocln, Epha2, Tns2,* and *Cyth3*; the angiogenic transcriptional regulators *Sox14, Foxo3, Klf4*, and *Gata2* (Fig. 8d). Other unique patterns are observed in regulators of MAP kinase (e.g., *Mapk4*), transporters (e.g., *Glr2*), the extracellular matrix (e.g., *Spock3*), and inflammation (e.g., *Il6*) (Additional file 1: Fig. S2a).

The effects of aging on BBB recovery results in a unique transcriptome and DNA methylome profile that includes the cell junction and polarity complex-encoding genes *Cldn5, Cldn11, Tjp2* and *Dlg5*; the angiogenic transcription factors *Ets2, Hes1,* and *Foxo1*; the Wnt signaling pathway genes *Fzd9, Wnt7a,* and *Wnt7b*; the Rho kinase pathway gene *Cdc42ep3*; the integrins *Itgb2, Itgb4* and *Itgb1;* and the transporters *Lrp10* and *Atp1a2*. The intriguing profile of aging poststroke BBB recovery is also characterized by a profound alteration in methylation and gene expression of regulators of endothelial to mesenchymal transformation (*Snai1, Smad6, Tgfbr2,* and *Sox9*) and epigenetic regulators (*Sirt2* and *Kdm6a*) (Fig. 8e and Additional file 1: Fig. S2b). It is important to highlight that the unique phenotype of the aging post-stroke BBB recovery is in part dependent on an altered profile of normal aging brain endothelial cells, as genes related to endothelial to mesenchymal transformation *Smad6* (p.adjust = 0.00011, q-value = 3.36E−09), and epigenetics, histone demethylase Kdm6a (p.adjust = 6.73E−15, q-value = 3.82E−06) are significantly upregulated at the transcription level in aged versus young non-stroke mice. Both changes were associated with hypomethylation (data not shown). This stresses that aging has profound effects on the BBB pre- and post-stroke recovery.

Thus, DNA methylation has an important role in BBB recovery, directing some of the critical processes involved in restoring the structural and functional BBB, such as angiogenesis, junctional proteins, establishment of polarity, actin cytoskeleton reorganization, extracellular matrix, as well as transporter system reestablishment. Nevertheless, DNA methylation could also contribute to the limited BBB recovery in young mice, mostly through the activation of specific signaling pathways (e.g., Rho GTPases), while in aging mice the limited BBB recovery could be due to the repression of structural protein expression (e.g., claudin-5), as well as activation of genes involved in endothelial to mesenchymal transformation, repression of angiogenesis, and epigenetic regulation.

## Discussion

Poststroke BBB recovery has gained more attention recently due to accumulating evidence linking poststroke BBB “status” with stroke outcomes [17, 19, 21, 23]. Prolonged poststroke BBB leakage can increase the risk for stroke recurrence, limit stroke recovery, and lead to poststroke complications including cognitive decline [3–5]. As the BBB possesses some capacity for recovery, it is critical to understand why the restoration process is limited and the role different pathways have in the recovery process. This study addresses two important questions regarding BBB poststroke recovery: (a) what is the profile of brain endothelial cells and the mechanisms involved in barrier recovery, and (b) how do epigenetic modifications induced by ischemic stroke direct the process of recovery? Our results highlighted that: (a) DNA methylation contributes to the control of transcript expression associated with BBB recovery in brain endothelial cells, (b) DNA methylation predominantly regulates genes that encode structural proteins (tight and adherens junction protein, actin cytoskeleton, cell polarity), extracellular matrix, transporter systems, angiogenesis (e.g., angiogenic transcription factors) and signaling pathways (Wnt/b-catenin and Rho GTPase), (c) aging/senescence plays a prominent role in altering DNA methylation and the transcriptomic profile in poststroke BBB recovery, (d) the existence of the limited poststroke BBB recovery and prolonged BBB leakage is due in part to the aberrant methylation and increased expression and potential activation of Rho GTPase, as well as shifting the profile of angiogenesis from physiological to pathological, and (e) aging is a profound factor of poststroke BBB injury/leakage triggering aberrant methylation and upregulation of genes encoding endothelial to mesenchymal transformation as well angiogenic repressors, restricting full BBB recovery. These findings are discussed below.

Ischemic stroke led to dynamic changes in DNA methylation, regulating widespread differential gene expression and modifying processes involved in injury and recovery [37–39]. The regulatory potential of DNA methylation is shaped by two opposing processes, the addition and removal of a methyl group at position five of cytosine in DNA [29, 30, 34, 36]. This results in transcriptional repression by preventing RNA polymerases from recognizing specific DNA regions or transcription upregulation by removing the “DNA methylation breaks”, respectively. The effect of DNA methylation on transcript expression is dependent on their location within the gene regions (promoter and/or gene bodies), with transcriptional changes typically dependent on methylation of multiple sites within a given gene [29, 30]. Global alterations in DNA methylation have been reported in stroke in several experimental and clinical studies, highlighting specific gene dynamics. For example, patients with a high risk for ischemic heart disease and stroke have hypomethylated Long Interspersed Nucleotide Element-1 (LINE-1) repeats, associated with increased circulating vascular cell adhesion molecule-1 (VCAM-1) levels [39]. Similarly, hypomethylation of TNF receptor-associated factor 3 (TRAF3), hypermethylation of thrombospondin-1 (THBS1), and increased DNMT3A activity are indicated as predictors of stroke outcome, as well degree of ischemic injury [38, 41]. So far, within the BBB, it has been reported that TIMP2 promoter hypermethylation or VCAM-1 promoter hypomethylation control BBB permeability and leukocyte recruitment [37, 40]. Brain and BBB recovery poststroke are associated with specific gene expression that controls the ongoing process of repair. DNA methylation appears to have important role, as the pattern of methylation changes at the global level. More important, however, are specific alterations in methylation patterns of genes encoding proteins directly involved in processes for re-establishing the barrier function, as these changes in methylation could repress (hypermethylation) or activate (hypomethylation) transcription.

In poststroke BBB recovery, angiogenesis is a pivotal process [12, 24, 56]. Starting 3–4 days after ischemic insult, intense remodeling of the vascular network occurs, involving brain endothelial cell proliferation and vessel sprouting that increases perfusion, predominantly in the peri-infarct area [12, 57]. Angiogenesis occurs over days and weeks and, depending on the degree, is positively correlated with stroke survival [12, 58]. Our results revealed DNA methylation as an apparent modifier of angiogenesis by altering transcription and upregulating expression of different angiogenesis regulators. That includes the transcription factors *Sox17*, a promotor of endothelial sprouting behavior and differentiation as well VEGFR2 expression in a cell-intrinsic manner, and ETS transcription factors family member *Erg*, that transactivate genes involved in key endothelial functions like survival, VEGF-angiogenic signaling pathways along with the VEGF receptor *Flt1*, [59–64]. Furthermore, altered DNA methylation regulates extracellular matrix remodeling, forming a more proangiogenic matrix by upregulating fibronectin (*Fn1*) and thrombospondin-4 (*Thbs4*) expression, and increasing expression of integrin β5, which is responsible for the cell–matrix interaction [65–67].

As the balance between proangiogenic and anti-angiogenic factors is critical for the formation of mature vessels, hypomethylation of angiogenic repressors like *Notch4* and its consequent upregulation is critical in part for limiting cell proliferation and vessel sprouting [68]. The next important step where DNA methylation contributes is blood vessel maturation, specifically in brain endothelial barrier formation. Promoter and gene body hypomethylation and subsequent transcript upregulation is seen in the group of genes regulating cell junction protein expression, such as Roundabout homolog 4 (*Robo4*) and the transcription factor SRY-Box 17 (*Sox17*) and *Erg* (regulator of VE-cadherin, claudin-5 expression), the structural tight junction protein and regulator of TJ complex stability occludin (*Ocln*), the actin cytoskeleton protein and stabilizer of TJ complex ezrin (*Ezr*), and the regulator of brain endothelial cell polarity cytohesin-3 (*Cyth3*), which controls Golgi complex structure and recruitment of junctional proteins [60, 69–71]. These data pinpoint that DNA methylation directly or indirectly (e.g., forming proangiogenic conditions) could regulate the plasticity and capacity of brain endothelial cells for vascular remodeling and barrier recovery.

A remaining question, however, is why BBB recovery is limited when the transcriptional and methylome profiles indicate activation of beneficial restoration processes. There are several potential causes of limited BBB recovery. The methylome and transcriptome profile showed that besides VEGF-mediated angiogenesis, there is also VEGF-independent angiogenesis. This is reflected by significant promoter hypomethylation and increased transcript expression of m6A demethylase alkB homolog 5 (*Alkbh5*) and thrombospondin-4 (TSP-4, *Thb4*), which acts as an accelerator of angiogenesis in response to TGF-β1, and contributes to cell- and disease-stage-specific effects of TGF-β [65, 72, 73]. Shifting the angiogenic process to a more pathological one through VEGF-independent angiogenesis might affect the full differentiation of endothelial cells, and particularly barrier maturation. In addition, the poststroke BBB methylome profile revealed promoter hypermethylation of glycine receptor 2 (*Glra1*) and repression of its expression. Repressing *Glra1* expression, an essential regulator of neurovascular/cerebrovascular remodeling and protector against post-ischemic injury, could potentiate injury processes at the BBB [74]. Furthermore, hypomethylation within the exons of the ephrin type-A receptor (*Epha2*) is reported to mediate brain endothelial cell TJ disruption in inflammatory conditions [75]. Other unfavorable factors for BBB recovery are the activation of the Rho GTPase RhoA and CDC42 effector protein 4 (*Cdc42ep4*), both indicated in regulating actin remodeling, stress fiber formation, and TJ complex disassembly, as well as atypical Map kinase *Mapk4*, the overexpression of which shifts cells to a proinflammatory phenotype [76–79]. Presumably, the balance, or lack thereof, between favorable and unfavorable processes/factors may predict the final outcomes of BBB poststroke recovery.

One important factor that predicts stroke and BBB recovery is aging. Aging causes a time-dependent decline in BBB properties, setting a variety of cellular functions at new levels. The epigenetic alterations largely reflect the aging-associated deleterious events. There is a loss of DNA methylation during aging and, with the advancement in age, genes such as estrogen receptor, insulin-like growth factor 2 (IGF2), and p16 are hypermethylated, with this abnormal DNA methylation leading to a heritable change [80]. Thus, the aged BBB could have its own profile of BBB recovery that reflects greater poststroke BBB leakage. Our data pinpoints two important findings. First, aberrant DNA methylation regulates some of the same transcripts as in young mice, like the upregulation of structural proteins ezrin (*Ezr*), actin related protein *Cnn3*, regulators of VEGF-associated angiogenesis (receptors *Kdr* and *Flt1*), the transcriptional factor *Erg,* as well as the *Ccnd1*/*Igfbp3* axis, which regulates the cell cycle. There is also hypomethylated/upregulated/ “activated” signaling pathways involved in re-establishment of the BBB, like the Wnt-beta-catenin signaling components, *Wnt7b* and *Fzd9* [81–83]. Furthermore, the aging BBB has similar limiting factors of the BBB recovery, such as the Rho GTPase *Rhoa* and *Cdc42ep1* overexpression during the poststroke recovery. However, the aging poststroke BBB DNA methylome and transcriptome profile is characterized by “unique” aberrant methylation in three categories: angiogenesis, structural proteins, and endothelial to mesenchymal transformation (EndMT). Although aging brain endothelial cells still possess angiogenic capacity, aging/senescence led to significant hypomethylation and upregulation of the angiogenic repressor transcriptional factor *Foxo1* and repression of two angiogenic factor transcription factors, *Klf8* and *Epha6* [84–86]. This could potentially induce more of an imbalance between pro- and anti-angiogenic factors and limit cerebrovascular network restoration. Recovery of the restrictive features of BBB is also disturbed, as aging brain endothelial cells have hypermethylation of the key TJ occlusion protein claudin-5, causing transcript downregulation. This finding is in agreement with some recent reports of decreased claudin-5 protein expression in aging mice [87]. Finally, the most profound changes are in the activation of EndMT. Both chronic inflammation and aging/senescence can transform brain endothelial cells towards a mesenchymal phenotype, with a loss of specific barrier endowed properties as well as functional/metabolic features [73, 88, 89]. Aberrant hypomethylation patterns associated with transcriptome upregulation occurred in some key players in EndMT, such as *Snai1*, *Sox9*, *Tgfb1* and *Tgfbr2,* that lead to brain endothelial cell dedifferentiation and limit barrier plasticity and capacity for recovery Thus, enhanced BBB poststroke injury with aging depends in part on epigenetic alterations that could limit BBB recovery. It is also important to underscore that the degree of BBB recovery in aging is in part dependent on the prior status of BBB as some of the genes (i.e., *Smad6, Sox14, Snai1*) are unique for both non-stroke and stroke aged groups, centering aging as a critical factor that determines the dynamics and outcome of poststroke BBB recovery.

### Study limitations

Designed to address the high cost and inefficiency of whole-genome bisulfite sequencing (WGBS), RRBS enriches for CpG dinucleotide-containing regions through enzymatic DNA digestion, yielding small DNA fragments comprising ~ 1% of the genome [90]. While other methylation profiling methods, such as WGBS and MeDIP-Seq, recognize DMRs with ≥ 2 CpG/100 bp and < 5 CpG/100 bp, respectively, RRBS only recognizes DMRs with ≥ 3 CpG/100 bp [91]. Comparatively, RRBS is biased toward CpG islands and promoter regions, with low coverage of repeat elements and enhancers [90–93]. While the function of non-promoter methylation is still being studied, current research suggests that methylation of enhancers, insulators, and low-density CpG regions (termed CpG deserts) has functional significance, indicating a relationship between their methylation and transcription [30, 94]. Likewise, aberrant methylation of CpG shores, defined as 2 kb from CpG islands, has been identified as a hallmark of specific disease states [95]. Ultimately, profiling the poststroke BBB methylome with RRBS focuses our analysis on promoter methylation and neglects methylation changes in low-density CpG regions, which also potentially contribute to transcriptional regulation.

Another limitation is inclusion of only one sex, male, in the study. Emerging evidence indicates that epigenetics has a major role in physiological sex differences and a broad range of disease susceptibility [96]. DNA methylation is considered a promising biomarker for aging and age-related diseases with several fold differences in methylation between male and female [97, 98]. Thus, future studies need to analyze the DNA methylation profile of the poststroke BBB in females to establish reliable the epigenetic profile of BBB recovery in stroke.

## Conclusion

In conclusion, altered DNA methylation patterns are present in BBB poststroke recovery, regulating the processes that restore the BBB property, while parallelly activating and regulating some factors contributing to BBB injury. With the opportunity to assess genome-wide DNA methylation patterns in poststroke recovery in different ages, we have defined the ongoing processes of BBB recovery (i.e., angiogenesis, barrier maintenance, signaling regulation via Rho GTPase and Wnt/beta catenin signaling, and EndMT) and their epigenetic (methylome) alterations that could limit the recovery process, resulting in profound BBB injury. This study offers new insights into the etiology of BBB and cerebrovascular decline after stroke and opens up new avenues for drug discovery and targeted therapies.

## Supplementary Information

**Additional file 1: Figure S1**. Comparison of poststroke BBB DNA methylome profile in young and old mice. Venn diagrams depicting the number of common (A) gene promoter DMRs and (B) gene body DMRs between young post-stroke TE mice and old post-stroke TE mice. Data represents statistically significant DMRs, and do not consider direction of change (e.g., hypermethylation or hypomethylation. Pearson correlation plot for common (C) gene promoter DMRs and (D) non-promoter DMRs, with x- and y-axes demonstrate percent methylation change for old post-TE stroke DMRs and young post-TE stroke DMRs, respectively. Statistically significant DMRs regulated in the same direction (e.g., hypermethylated in both groups) are blue, while statistically significant DMRs regulated in opposite directions (e.g., hypermethylated in aging post-TE stroke and hypomethylated in young post-TE stroke) are red. Methylation of gene promoters had a statistically significant, but weak positive correlation between experimental groups (R = 0.0188, p = 0.00055). Non-promoter methylation does not correlate across experimental groups (R = 0.0018, p = 0.5227). Summary of gene over-representation analysis with DMRs common to both experimental groups, demonstrating enriched GO terms for (E) DMRs regulated in same direction (e.g., hypermethylated in both groups) or (F) opposite direction (e.g., hypermethylated in aging post-TE stroke and hypomethylated in young post-TE stroke). Enriched GO terms were selected based on their statistical significance (q value < 0.05) and relevance to endothelial cell biology.

**Additional file 2: Figure S2**. Unique DEGs with altered DNA methylation. Tables depicting unique DEGs with altered DNA methylation in BBB of (A) young mice and (B) aging mice. Genes were selected based on their relevance to endothelial cell biology and classified based on biological function. Categories for both young and aging post-TE stroke include transporters and receptors, extracellular matrix and integrins, and inflammation. Categories unique to aging post-TE stroke include epigenetics and protein sorting.

## Data Availability

The datasets presented in this study can be found in online repositories. The repository is National Center for Biotechnology Information (NCBI) and accession number PRJNA918370 and PRJNA833447. https://www.ncbi.nlm.nih.gov/bioproject/PRJNA918370.
