# Supplementary figures and images for: Epigenetics and stroke: role of DNA methylation and effect of aging on blood–brain barrier recovery
Source: Fluids Barriers CNS. 2023 Feb 28;20:14. doi: 10.1186/s12987-023-00414-7 (PMC9972738; doi:10.1186/s12987-023-00414-7)

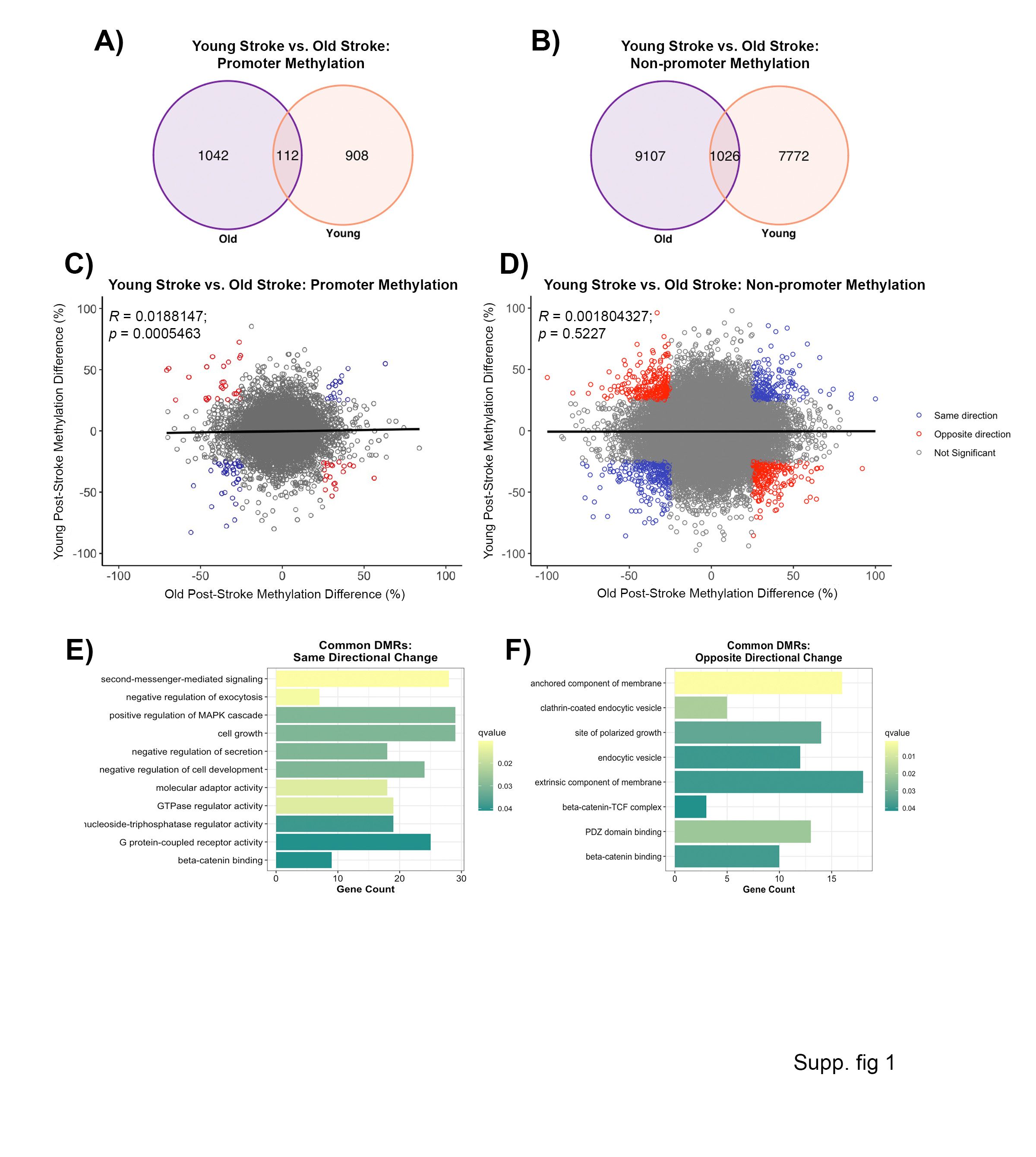

Supplement: Supplementary file 1 — Additional file 1: Figure S1. Comparison of poststroke BBB DNA methylome profile in young and old mice. Venn diagrams depicting the number of common (A) gene promoter DMRs and (B) gene body DMRs between young post-stroke TE mice and old post-stroke TE mice. Data represents statistically significant DMRs, and do not consider direction of change (e.g., hypermethylation or hypomethylation. Pearson correlation plot for common (C) gene promoter DMRs and (D) non-promoter DMRs, with x- and y-axes demonstrate percent methylation change for old post-TE stroke DMRs and young post-TE stroke DMRs, respectively. Statistically significant DMRs regulated in the same direction (e.g., hypermethylated in both groups) are blue, while statistically significant DMRs regulated in opposite directions (e.g., hypermethylated in aging post-TE stroke and hypomethylated in young post-TE stroke) are red. Methylation of gene promoters had a statistically significant, but weak positive correlation between experimental groups (R = 0.0188, p = 0.00055). Non-promoter methylation does not correlate across experimental groups (R = 0.0018, p = 0.5227). Summary of gene over-representation analysis with DMRs common to both experimental groups, demonstrating enriched GO terms for (E) DMRs regulated in same direction (e.g., hypermethylated in both groups) or (F) opposite direction (e.g., hypermethylated in aging post-TE stroke and hypomethylated in young post-TE stroke). Enriched GO terms were selected based on their statistical significance (q value < 0.05) and relevance to endothelial cell biology. [file 12987_2023_414_MOESM1_ESM.jpg]

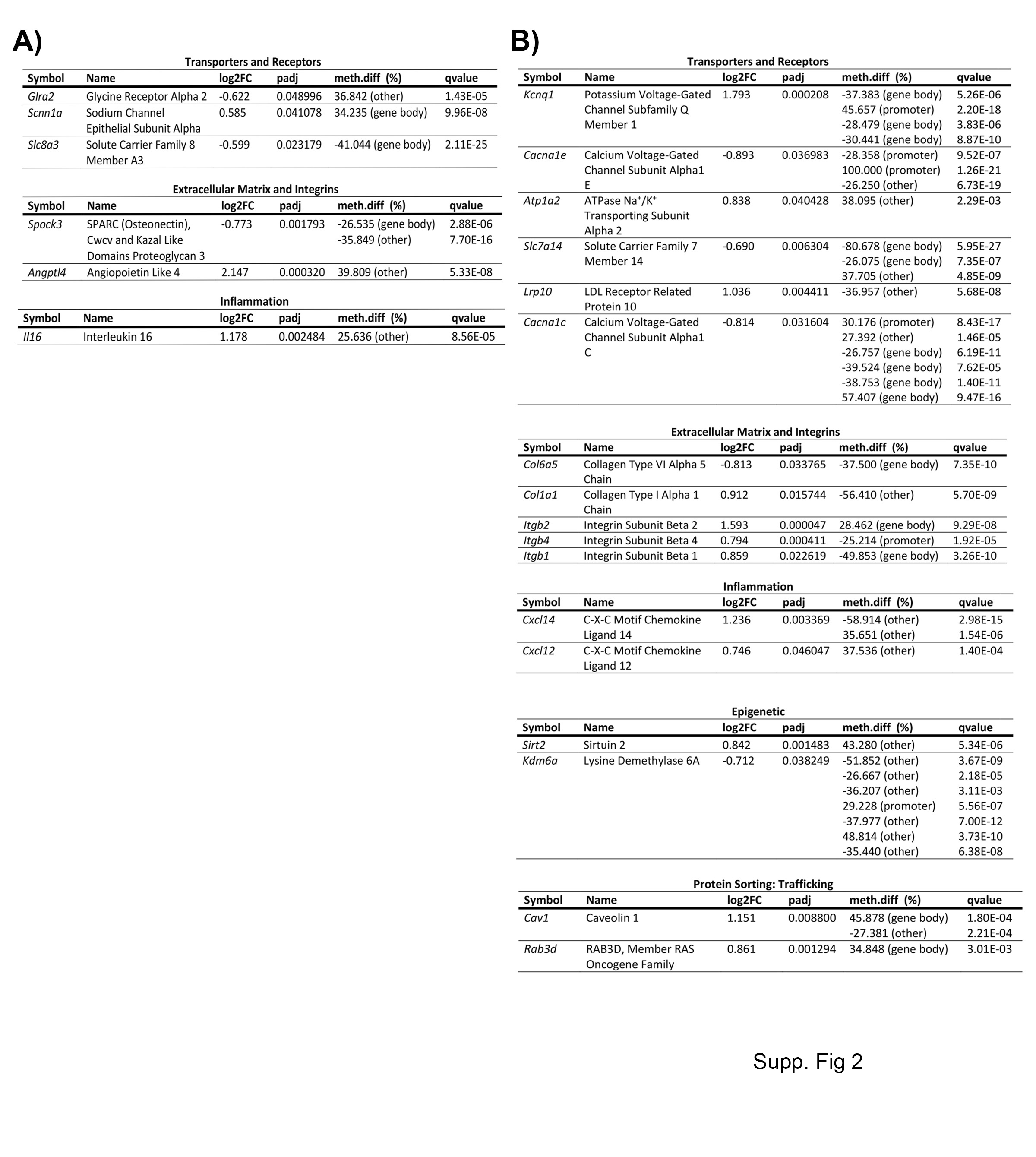

Supplement: Supplementary file 2 — Additional file 2: Figure S2. Unique DEGs with altered DNA methylation. Tables depicting unique DEGs with altered DNA methylation in BBB of (A) young mice and (B) aging mice. Genes were selected based on their relevance to endothelial cell biology and classified based on biological function. Categories for both young and aging post-TE stroke include transporters and receptors, extracellular matrix and integrins, and inflammation. Categories unique to aging post-TE stroke include epigenetics and protein sorting. [file 12987_2023_414_MOESM2_ESM.jpg]
